# Integration Analysis of Single‐Cell Multi‐Omics Reveals Prostate Cancer Heterogeneity

**DOI:** 10.1002/advs.202305724

**Published:** 2024-03-14

**Authors:** Xiaojie Bian, Wenfeng Wang, Mierxiati Abudurexiti, Xingming Zhang, Weiwei Ma, Guohai Shi, Leilei Du, Midie Xu, Xin Wang, Cong Tan, Hui Sun, Xiadi He, Chenyue Zhang, Yao Zhu, Min Zhang, Dingwei Ye, Jianhua Wang

**Affiliations:** ^1^ Department of Urology Fudan University Shanghai Cancer Center Department of Oncology Shanghai Medical College Fudan University Shanghai 200032 China; ^2^ Cancer Institute Shanghai Urological Cancer Institute Fudan University Shanghai Cancer Center Department of Oncology Shanghai Medical College Fudan University Shanghai 200032 China; ^3^ Department of Urology Shanghai Pudong New Area Gongli Hospital Shanghai 200135 China; ^4^ Department of Pathology Fudan University Shanghai Cancer Center Shanghai 200032 China; ^5^ Department of Cancer Biology Dana‐Farber Cancer Institute Boston MA 02215 USA; ^6^ Department of Biological Chemistry and Molecular Pharmacology Harvard Medical School Boston MA 02115 USA; ^7^ Department of Integrated Therapy Fudan University Shanghai Cancer Center Shanghai 200032 China; ^8^ Pediatric Translational Medicine Institute and Pediatric Congenital Heart Disease Institute Shanghai Children's Medical Center Shanghai Jiao Tong University School of Medicine Shanghai 200127 China

**Keywords:** CD8^+^T cell, heterogeneity, prostate cancer, single‐cell RNA sequencing, spatial transcriptomics, the tumor microenvironment

## Abstract

Prostate cancer (PCa) is an extensive heterogeneous disease with a complex cellular ecosystem in the tumor microenvironment (TME). However, the manner in which heterogeneity is shaped by tumors and stromal cells, or vice versa, remains poorly understood. In this study, single‐cell RNA sequencing, spatial transcriptomics, and bulk ATAC‐sequence are integrated from a series of patients with PCa and healthy controls. A stemness subset of club cells marked with *SOX9^high^AR^low^* expression is identified, which is markedly enriched after neoadjuvant androgen‐deprivation therapy (ADT). Furthermore, a subset of *CD8^+^CXCR6^+^* T cells that function as effector T cells is markedly reduced in patients with malignant PCa. For spatial transcriptome analysis, machine learning and computational intelligence are comprehensively utilized to identify the cellular diversity of prostate cancer cells and cell‐cell communication in situ. Macrophage and neutrophil state transitions along the trajectory of cancer progression are also examined. Finally, the immunosuppressive microenvironment in advanced PCa is found to be associated with the infiltration of regulatory T cells (Tregs), potentially induced by an *FAP^+^* fibroblast subset. In summary, the cellular heterogeneity is delineated in the stage‐specific PCa microenvironment at single‐cell resolution, uncovering their reciprocal crosstalk with disease progression, which can be helpful in promoting PCa diagnosis and therapy.

## Graphical abstract

Understanding the cellular heterogeneity within the prostate cancer (PCa) microenvironment is crucial for unraveling the mechanisms that drive tumor progression. Bian et al. integrate single‐cell RNA sequencing, spatial transcriptomics, and bulk ATAC‐sequence analysis to provide a multi‐dimensional view of the complex ecosystem in the tumor microenvironment. Findings from this research can provide novel perspectives when treating advanced prostate cancer.

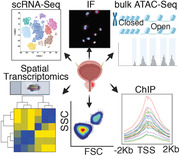

## 1 Introduction

Prostate cancer (PCa) is the most common malignancy in men worldwide.^[^ ^1^ ^]^ PCa is a heterogeneous disease with different biological subtypes and different prognostic impacts. Androgen‐deprivation therapy (ADT) is the standard therapy for metastatic PCa.^[^ ^2^ ^]^ Although ADT is initially effective in treating metastatic PCa, luminal epithelial cells undergo redifferentiation or transdifferentiation and are, therefore, resistant to this treatment. Previous studies have shown that late relapse often arises from Darwinian selection in genetically heterogeneous cancer cell populations. In addition to the Darwinian selection model, prostate cancer stem cells (CSCs) are another source of biochemical recurrence after ADT. *Guo et al.* reported a prostate stem/progenitor cell subset, marked with *PSCA*, *CK4*, and *TACSTD2* expression, that exhibited a greater capacity for organoid formation in vitro and prostate epithelial duct regeneration in vivo, suggesting a potential cellular origin of prostate cancer.^[^ ^3^ ^]^ Another report identified a subpopulation of luminal progenitors with *LY6D* expression that were intrinsically resistant to castration with a bilineage gene expression pattern,^[^ ^4^ ^]^ indicating that there are pre‐existing CSCs or progenitors that contribute to late relapses.

Two rare subpopulations with unique transcriptional profiles have been reported in independent studies.^[^ ^5^ ^]^ One subset, named “club cell,” is characterized by the expression of *PIGR*, *MMP7*, and *CP*. Interestingly, patient PIGR‐enriched extracellular vesicles drive cancer stemness, tumorigenesis, and metastasis by activating PDK1/Akt/GSK3β/β‐catenin signaling cascades in hepatocellular carcinoma,^[^ ^6^ ^]^ while *MMP7* is closely related to the EMT process, promoting invasion and migration of cancer cells.^[^ ^7^ ^]^ The other subset, termed “hillock cell,” has a transcriptomic profile very close to club cell but uniquely expresses *KRT5* and *KRT13*. Although previous studies have shown that club and hillock cells are enriched in the urethra and periurethral prostate zones,^[^ ^5a^ ^]^ the functions of these cells in the prostate are poorly understood.

Tumor‐infiltrating immune cells in the PCa tumor microenvironment (TME) represent another factor that affects prostate cancer progression. The composition of tumor‐infiltrating immune cells is highly heterogeneous, and recruitment/activation of antigen‐presenting cells (APCs), such as dendritic cells (DCs), monocytes, or “M1” macrophages, in treated prostate tissues may contribute to local T cell activation.^[^ ^8^ ^]^ Tumor‐infiltrating regulatory T cells or the recruitment/activation of tumor‐associated macrophages (TAMs) could attenuate antigen recognition, leading to decreased *CD8^+^* T cell cytotoxic function or proliferation.^[^ ^9^ ^]^ Emerging data show that tumor‐infiltrating neutrophils play a dual role in cancer progression.^[^ ^10^ ^]^ Similar to macrophages, tumor‐infiltrating neutrophils undergo a transition from “N1” polarization (associated with antitumor functions) to “N2” polarization (promoting cancer development and progression). A recent study found that castration‐mediated IL‐8 expression promotes prostate cancer progression via myeloid infiltration, mainly infiltrating dysfunctional neutrophils.^[^ ^11^ ^]^ These studies suggest that elucidating the composition and functional state of immune cells would be helpful for treating PCa.

In addition to tumor‐infiltrating immune cells, stromal cells, particularly activated fibroblasts (cancer‐associated fibroblasts, CAFs), play a central role in breast and pancreatic carcinoma carcinogenesis.^[^ ^12^ ^]^ Previously, we identified two distinct fibroblast clusters representing myofibroblastic or ECM‐associated phenotypes in the PCa microenvironment.^[^ ^13^ ^]^ Functional heterogeneity of mouse prostate stromal cells revealed by scRNA‐seq identified an inflammatory prostate CAF subset (*SCA‐1^+^CD90^low^* fibroblasts) expressing ECM‐related genes as well as genes encoding cytokines, chemokines, and complement components (*Ccl2*, *Ccl7*, *Ccl11*, *Cxcl1*, *Cxcl2*, and *C3*),^[^ ^14^ ^]^ indicating that reclassification of the fibroblast subsets should be further optimized.

In this study, we hypothesized that reciprocal crosstalk between tumor and interstitial cells may reshape the tumor microenvironment and directly affect tumor progression. Comprehensive analysis of the epithelia, tumor‐infiltrating immune cells, and stromal cells using scRNA‐seq revealed a dynamic state transition under different biological conditions in vivo. We found evidence of lineage plasticity of *SOX9^High^AR^low^* club cells in patients undergoing ADT and further report that the Wnt/β‐catenin pathway activation is responsible for lineage plasticity after ADT. ADT‐induced cell death boosts immune response, recruits APCs (*IL1B^+^NLRP3^+^* macrophages), and promotes *CD8^+^* T cell activation. In addition, we identified a new tumor‐associated macrophage subset named TAM‐C2, marked without *CD86* expression (an important co‐stimulatory molecule for *CD8^+^* T cell activation), almost exclusively in patients with PCa, which is partly responsible for disease progression. Finally, we revealed that abundant FAP^+^ fibroblasts in advanced PCa and neuroendocrine PCa (NE) may promote Treg differentiation, further creating an immunosuppressive microenvironment that, in turn, helps tumor cells escape immune surveillance.

## 2 Results

### 2.1 Epithelia, Lymphocytes, and Myeloid Cells are the Main Cell Types in the PCa Microenvironment

**Figure 1:**
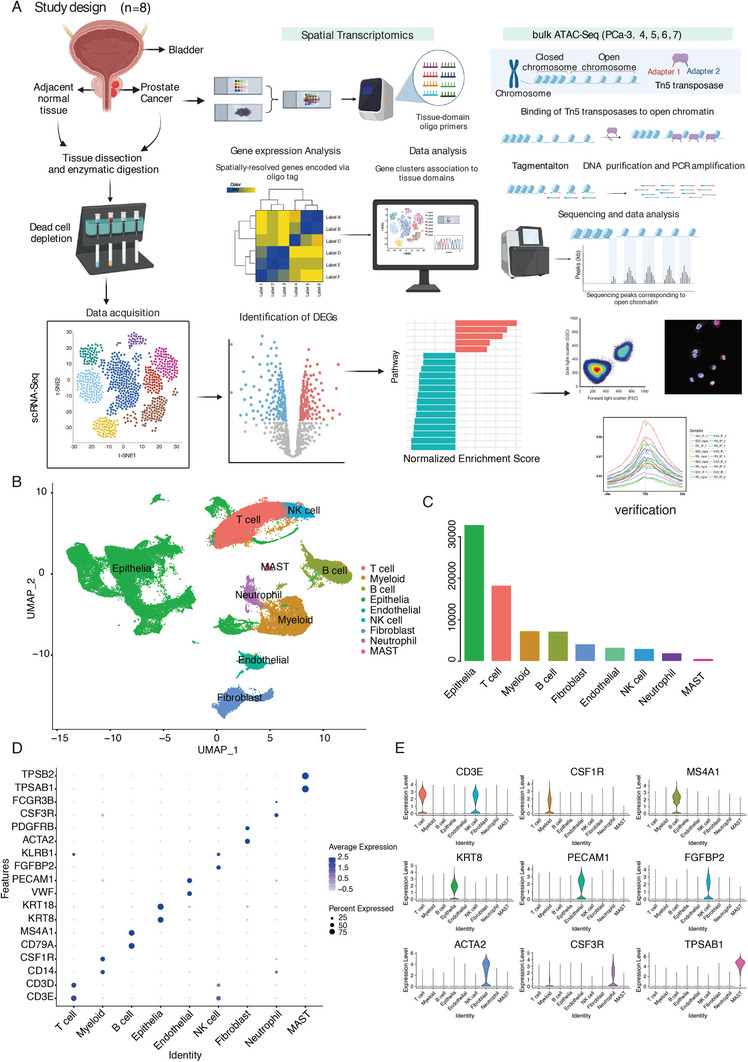
Study design and overview of the PCa microenvironment. A) Illustration of the study design. 7 PCa tissues paired with one normal prostate as control performed ScRNA‐Seq as well as spatial transcriptomics. Subsequent bioinformatics analysis, including cell type annotation, differential gene expression visualization, pathway enrichment analysis, and cell‐cell communication are used to dissect the ecosystem of the prostate under normal or unhealthy conditions. We comprehensively utilized multiple molecular biology experiments to depict the dynamic changes in the PCa evolution. Figures created with BioRender (https://biorender.com/). B) UMAP plot showed PCa microenvironment composed of 9 major cell types. C) The absolute number of the 9 major cell types in PCa. Epithelia and T cells rank in the top 2 positions among the 9 cell types. D,E) Visualizing marker gene expression in dot plot (D) or violin plot (E).

To dissect the diversity of cell composition and transcriptional states of cells in PCa specimens, we performed single‐cell analysis of PCa tissues from radical prostatectomy (RP) specimens with six localized PCa (PCa‐2‐7) samples, one neuroendocrine prostate cancer (NEPC) sample (PCa‐1), and one normal prostate (PCa‐8) sample as control. Hematoxylin and eosin (H&E) and immunohistochemical (IHC) staining were used to identify the pathological types of PCa (Figure S1A–D and Table S1, Supporting Information). All the samples were paired using spatial transcriptomics. In total, 77 661 cells were analyzed and nine different major cell types were identified, marked by specific gene expression profiles (see “Experimental Section,” **Figure** **1**A–E).

Cells in the merged dataset were annotated as epithelia (*KRT8*, *KRT18*), T cell (*CD3E*, *CD3D*), myeloid cell (*CD14*, *CSF1R*), B cell (*CD79A*, *MS4A1*), fibroblast (*ACTA2*, *PDGFRB*), endothelial cell (*PECAM1*, *VWF*), NK cell (*FGFBP2*, *KLRB1*), neutrophil (*CSF3R*, *FCGR3B*), and mast cell (*TPSAB1*, *TPSB2*), based on established marker genes (Figure 1D,E). Unsurprisingly, epithelial cells constituted the predominant cell type in all samples, except the normal control (Figure S1E, Supporting Information).

### 2.2 Delineation of Epithelial Cell Subset Heterogeneity and Cancer Epithelia Evolution

**Figure 2:**
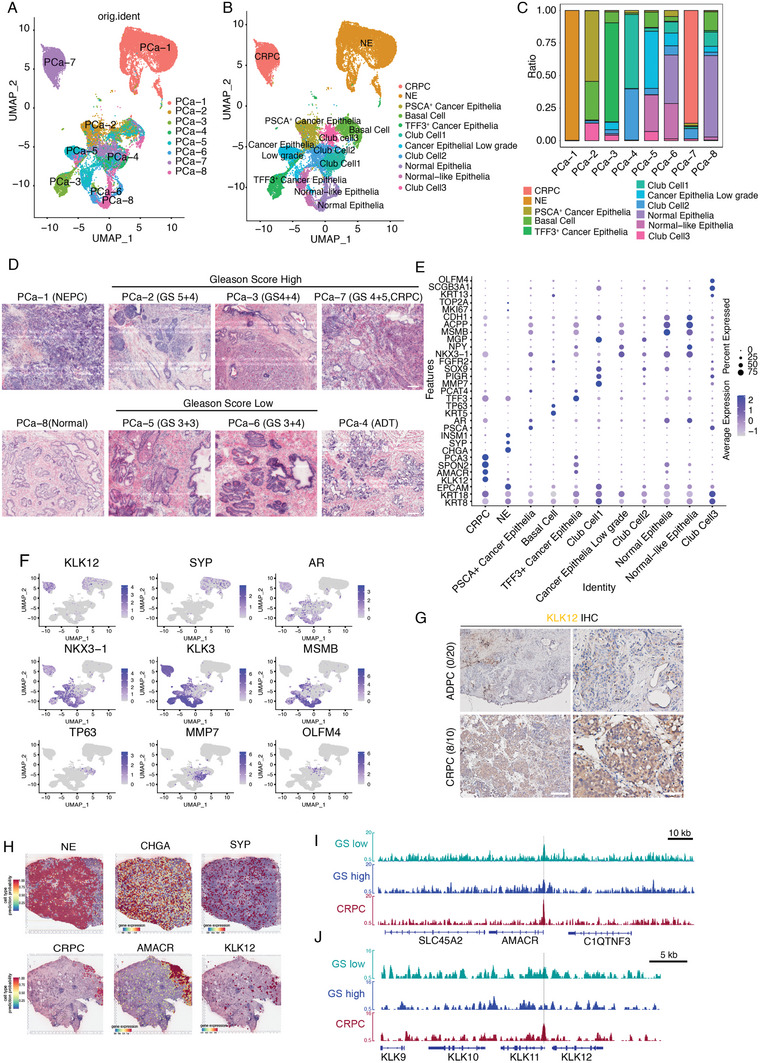
Epithelial cell subsets exhibit extreme heterogeneity and chromatin accessibility of cancer epithelia changes dramatically as disease progresses. A,B) Visualizing epithelial subsets by original identity (A) or subpopulation (B). C) Stack graph shows the epithelial subset composition in each patient. The cellular composition of NE patient is predominantly composed of neuroendocrine cells, while the PCa‐7 patient is predominantly composed of CRPC epithelial cells. D) H&E staining illustrated the cellular morphology in different patients with different GS. PCa‐8 is a normal prostate. Scale bar: 50 µm. E,F) Visualizing the marker genes E) expression in dot plot or F) expression pattern in feature plot. G) IHC staining showed KLK12 robust expressed in CRPC patients (8 out of 10 CRPC patients are KLK2 positive) while having little or no expression in all 20 HSPC patients. Scale bar: 50 µm. H) Spatial transcriptomics analysis showed the NEPC patient robustly expressed CHGA and SYP, while the canonical prostate adenocarcinoma marker AMACR (p504s), expressed significantly higher in CRPC patients. I,J) Compared to adenocarcinoma, the CPRC patient with more accessible chromatin at *AMACR* and *KLK11* locus.

Epithelial cells (*N*  =  32 741) were identified based on KRT8 and KRT18 expression levels. They were clustered into 11 subgroups using batch correction and unsupervised clustering analysis. Notably, the luminal epithelial cells were highly heterogeneous. Each cluster mainly originated from the corresponding patient (**Figure** **2**A–C). In comparison, basal cells, characterized by the expression of KRT5 (CK5) and transcription factor TP63 (P63), formed a distinct cluster collectively contributed by different patients (Figure 2A,B; Figure S2A, Supporting Information).

To investigate whether the single‐cell transcriptome aligned with the histological features of PCa, we performed Gleason grading for each patient's H&E staining (Figure 2D). The evaluation revealed a typical neuroendocrine prostate cancer pattern (PCa‐1), adenocarcinoma with Gleason scores ranging from 6 to 9 (PCa‐2‐7), and a normal donor (PCa‐8).

The NE sample, distinct from the others, highly expressed unique gene markers, such as CHGA and SYP. Meanwhile, NKX3‐1, MSMB, and ACPP were highly expressed in normal luminal cells (Figure 2E,F). The canonical prostate adenocarcinoma markers AMACR (p504s) and KLK3 (PSA) were robustly expressed in the castration‐resistant prostate cancer (CRPC) epithelia (Figure 2E,F). Notably, KLK12, a member of the kallikrein family that is barely expressed in other samples, was markedly upregulated in patients with CRPC (Figure 2F). To investigate the activation of KLK12 expression as a unique marker for CRPC, we compared its expression pattern in patients with hormone‐sensitive PCa (HSPC) using IHC staining. KLK12 staining was positive in 8 of the 10 patients with CRPC, while none of the 20 patients with HSPC showed positive staining (Figure 2G). We also analyzed the main cell types and visualized the spatial transcriptomic data of patients with NEPC (PCa‐1) and CPRC (PCa‐7). Spatial transcriptomic analysis showed that NEPC patients robustly expressed CHGA and SYP, whereas AMACR (p504s) and KLK12, unique markers for CRPC, were considerably higher in CRPC patients (Figure 2H).

Interestingly, scRNA‐seq also revealed transcriptomic features that were hardly distinctive in histological analysis. For example, H&E staining suggested that most of the epithelia of PCa‐6 were “normal epithelia” (Figure 2D). However, scRNA‐seq showed that ≈20% of epithelial PCa‐6 cells closely resembled cancer cells (Figure 2C). Another subset of luminal epithelia showed a transcriptome similar to that of normal epithelial cells. But they also had a characteristic gain expression of Neuropeptide Y (NPY). Subsequently, we annotated this cluster “Normal‐like Epithelia” subset and found that it mainly appeared in patients with low Gleason score (GS) (Figure 2C,E). These results suggest that transcriptomic changes may occur before the development of obvious histological patterns in prostate cancer.

To further distinguish tumor cells from normal cells, we used the “inferCNV” package (https://github.com/broadinstitute/inferCNV) to estimate copy number variations (CNVs) from our scRNA‐seq data. For the inferCNV analysis, we enrolled the most aggressive type of prostate cancer epithelia, NEPC (abbreviated as NE), the less aggressive malignant tumor cells (abbreviated as Cancer), the non‐invasive normal epithelial cells (abbreviated as Normal), and the precancerous lesion normal‐like epithelial cells (abbreviated as PIN). We compared whole‐genome copy number variation (CNV) among these cells (Figure S2B, Supporting Information). We found that NE cells showed the greatest CNV difference compared to the other cell types. In addition, a subset of cancer cells was clustered between PIN and NE cells. In contrast, the normal epithelial cells did not show noticeable differences form the reference cells, which indicates that the epithelial subsets we nominated “Normal,” “Normal‐like,” and “Tumor” are unbiased.

Common genetic alterations, including *FOXA1* mutations, gain of the androgen receptor gene (*AR* amplification or mutation), and structural variants such as loss of *NKX3.1* (8p21) and *PTEN* (10q23), account for prostate carcinogenesis. In addition, combinations of deletions of the tumor suppressors *TP53* and *RB1* are believed to promote prostate cancer resistance to a spectrum of therapeutics and ultimately treatment‐emergent NEPC. Therefore, we also evaluated the genetic hotspots among these sub‐clusters using the z‐score. We found that the “NE cells” display predominant *RB1* and *CDKN1B* loss (Figure S2C, Supporting Information) as well as *MYC* amplification (Figure S2D, Supporting Information). Additionally, we found that the “Cancer cells” possessed the highest *KLK3* score, a relatively high *AR* score, and a lower *NKX3‐1* score compared to the other subsets (Figure S2D, Supporting Information). These results, together with the inferCNV analysis,CV the overall high robustness of our epithelial subset nomenclature.

Changes in chromatin accessibility occur during development and cancer. To investigate the heterogeneity of luminal cells in chromatin accessibility, we compared genome‐wide chromatin accessibility between low‐ and high‐GS samples using an assay for transpose‐accessible chromatin (ATAC) sequencing and bulk RNA sequencing. ATAC‐Seq results revealed that with PCa progression, transcriptional activation of AR target genes, such as *KLK2*, *KLK3*, and *FKBP5*, changed remarkably and correlated with the AR transcriptional program (Figure S3A–C, Supporting Information), whereas patients with CRPC had more accessible chromatin at the *KLK11* and *AMACR* locus (Figure 2I,J).

To further validate that these target genes were unique AR targets, we analyzed a pan‐cancer ATAC‐seq dataset (PANCAN, https://gdc.cancer.gov/about‐data/publications/pancanatlas), which contains multilevel genomic data from 404 individual samples from a variety of cancer types. The well‐known AR target genes *TMPRSS2* and *FKBP5* show enhanced chromatin accessibility, as assessed by ATAC signals, in prostate cancer samples (TCGA‐PRAD). In addition, the target genes identified in our study, including *KLK2*, *KLK3*, *KLK11*, and *KLK12*, showed consistent ATAC peak enrichment in prostate samples (Figure S3D, Supporting Information). These results, together with our findings, revealed the heterogeneity of epithelial cells with PCa progression.

### 2.3 Targeted therapy generates distinct SOX9^High^ stem cell‐like state epithelia

**Figure 3:**
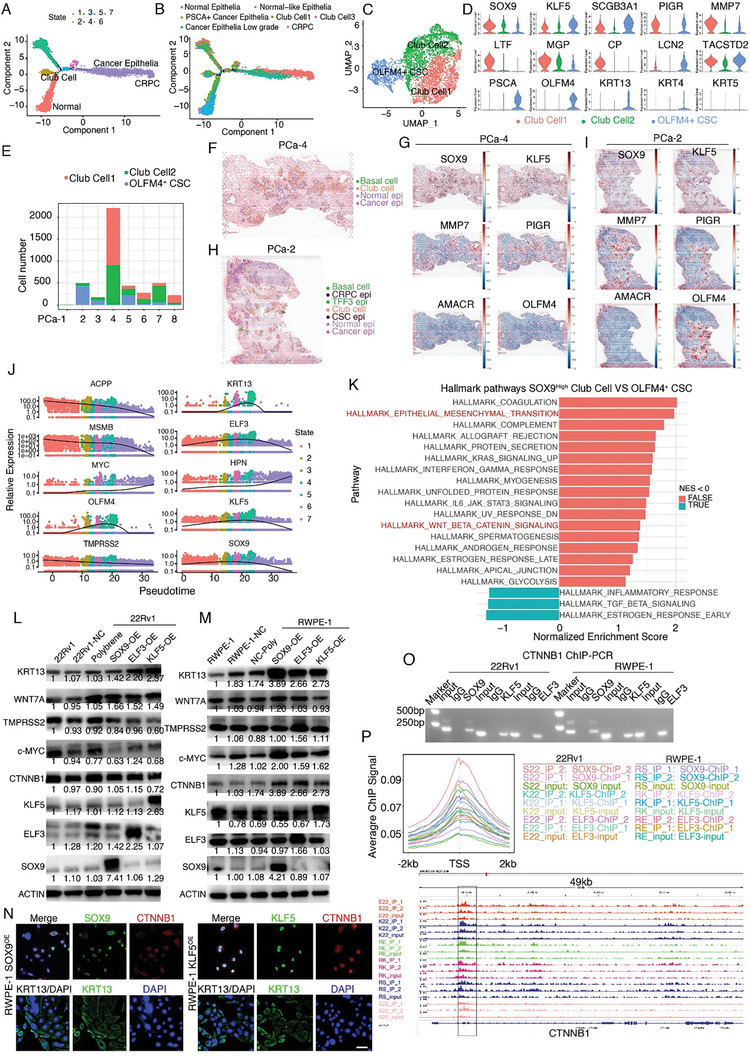
Targeted therapy generates distinct *SOX9^High^* stem cell‐like state epithelia. A,B) Pseudotime cell trajectories analysis using Monocle 2 to study the epithelial cellular dynamic changes as PCa progression. A) Visualization in state or B) Seurat cluster. Club cells located in the branching point of the pseudo‐time trajectory map. C,D) Club cell subsets illustrated in C) UMAP plot and D) in violin plots. PIGR, MGP, and MMP7 are canonical club cell markers. The OLFM4^+^CSCs showed a characteristic expression of PSCA and OLFM4. E) Stack graph showed the SOX9^High^ epithelial subset predominantly contributed by PCa‐4 patients. F,I) Depict the epithelial subset composition in situ. Overview of the epithelial composition in F) PCa‐4 and H) PCa‐2 patients. Spatial transcriptomics profile revealed the *SOX9* ^high^ Club cells and OLFM4^+^CSCs share a similar transcriptomic landscape but separate in spatial position. J) Pseudotime trajectory analysis results indicated SOX9 and KLF5 over‐activation might be the driver of normal luminal cell dedifferentiation to cancer stem cells. MYC may be another accelerator that fuels luminal cells to differentiate into cancer epithelial cells. K) Hallmark pathways analysis suggested the SOX9^High^ epithelial subset enrichment in HALLMARK_EPITHELIAL_MESENCHYMAL_TRANSITION and HALLMARK_WNT_BETA_CATENIN_SIGNALING pathway. L,M) Western blot results suggested that SOX9 or KLF5 over‐activation confers 22Rv1 and RWPE‐1 cell lines with stemness. MYC and CTNNB1 were significantly activated after SOX9 or KLF5 over‐activation in RWPE‐1 cell line. Western blot analysis of cell lysates was performed using antibodies against KRT13, WNT7A, TMPRSS2, MYC, CTNNB1, KLF5, ELF3, SOX9, and ACTIN. N) SOX9 or KLF5 over‐activation drives nuclear β‐catenin localization. Scale bar: 50 µm. O) ChIP‐PCR and P) ChIP‐Seq results suggested CTNNB1 is the direct target of SOX9 and KLF5.

To better understand the dynamic changes in epithelial cell states, we performed a trajectory analysis of epithelial subpopulations. NE cells showed a distinct transcriptome profile that was disconnected from that of other luminal cells, suggesting a unique origin (Figure S4A–C, Supporting Information). Therefore, we removed NE cells and reconstructed the trajectory map, which showed two branches that mimicked PCa oncogenesis and evolution (**Figure** **3**A,B; Figure S4D,E, Supporting Information). Interestingly, club cells marked with *PIGR* and *SCGB3A1* were located at the branching point of the pseudo‐time trajectory map (Figure 3A,B). To further validate the robustness of the trajectory analysis result, we selected the same cells used for Monocle 2 analysis for re‐clustering with the new package Monocle 3^[^ ^15^ ^]^ (http://cole‐trapnell‐lab.github.io/monocle‐release/). The re‐analysis trajectory results using Monocle 3 were very similar to those using Monocle 2; however, the CRPC subset was disconnected from the other subsets (Figure S4F,G, Supporting Information). These results indicated the robustness and reliability of the inferred trajectories.

Club cells are a relatively rare epithelial population thought to have cancer stem cell features. Unsupervised clustering analysis revealed three club cell subpopulations. The MGP^+^SCGB3A1^+^ cells resembled club cells found in normal prostate tissues (club cell 2). The OLFM4^+^KRT13^+^ club cells (club cell 3) co‐expressed the luminal C cell markers PSCA, TACSTD2, and KRT4 (CK4); hence, we termed this cluster OLFM4^+^CSC (Figure 3C,D). Intriguingly, we found that the SOX9^High^KLF5^+^ club cell population (club cell 1) was primarily enriched in a patient who underwent ADT (PCa‐4) (Figure 3E, Figure S4H–J, Supporting Information).

To further validate these findings, we first checked the published data, which included eight PCa patients (four hormonally intact and four ADT‐treated)^[^ ^16^ ^]^ and validated the club cell signatures in these patients. We found that the signatures of SOX9^high^AR^low^ club cells were markedly enriched in ADT‐treated patients compared to those in the hormonally intact group (Figure S5A–D, Supporting Information). Despite the increasing trend in the ADT‐treated group, the club cell ratio was not significantly different between the hormonally intact and control ADT‐treated groups (Figure S5E,F, Supporting Information). It is well known that basal cell loss is a recognized histopathological feature in diagnosing PCa. Given the significance of this observation, we investigated whether the presence of excess basal cells in this dataset overshadows the primary issue under investigation. To address this concern, we deliberately eliminated basal cells from our analysis and reassessed the club cell ratio. Accordingly, we found that the club cell ratio considerably increased in the ADT‐treated group compared to that in hormone‐naïve patients. This suggests that ADT induced luminal cell death but had little to no effect on club cells.

Furthermore, we performed IHC staining in 11 hormonally intact and 16 ADT‐treated patients (ADT exposure time: 1 week to 14 months, median time: 4.3 months, Table S2 (Supporting Information) patient information for club cells, Supporting Information). Our results demonstrate that the residual morphologically “normal” cells were characterized with negative AR but expressed high levels of MMP7 and LTF (Figure S6A,B, Supporting Information), indicating that they are indeed club cells. The statistical results showed that the club cell ratio increased remarkably during the ADT exposure period (Figure S6C, Supporting Information), as reported previously. We also explored the spatial distribution of epithelial cells using a machine learning‐based annotation method (Figure 3F–I; Figure S7A–E, Supporting Information). Spatial transcriptomics confirmed that most epithelial cells in the ADT‐treated patient (PCa‐4) were club cells (Figure 3F,G).

To investigate the transcription factors that may drive differentiation of epithelial cells, we performed SCENIC^[^ ^17^ ^]^ analysis. The SCENIC results suggest that normal and cancer epithelia share canonical luminal transcription factors (TFs) such as *HOXB13*, *GATA2*, *AR*. *MYC*, and *FOXA1*, which are often amplified or abnormally activated during oncogenesis. These factors were found to be considerably upregulated in the cancer epithelial cells (Figure S7F, Supporting Information). Meanwhile, the identification of transcriptional changes in tumors in the transition state (mainly SOX9^High^ club cell 1 and OLFM4^+^CSC epithelial cell club cell 3) suggests that TFs related to cell stemness, such as *SOX9*, *KLF5*, and *KLF4*, may govern the transition state. Several studies have reported the emergence of highly plastic cell states during ADT.^[^ ^2^, ^18^ ^]^ Pseudo‐time trajectory analysis indicated that SOX9 and KLF5 overactivation might be the drivers of normal luminal cell dedifferentiation into cancer stem cells. MYC, the aforementioned proto‐oncogene, may be another accelerator that fuels luminal cell differentiation into epithelial cancer cells (Figure 3J). We hypothesized that residual SOX9^High^ club cells may have an optional origin that accounts for PCa biochemical recurrence.

To identify gene signature differences between SOX9^High^ Club cells and OLFM4^+^ CSC, we performed pseudo‐bulk differential gene expression analysis on these two populations. The gene ontology analysis revealed genes involved in epithelial‐mesenchymal‐transition, and the Wnt/β‐Catenin pathway was enriched in SOX9^High^ club cells, indicating that SOX9^High^ club cells have some propensity to develop stemness characteristics or initiate metastasis (Figure 3K).

To investigate whether SOX9 and KLF5 are drivers of cancer stemness, we overexpressed these two transcription factors in vitro using the prostate cancer cell line 22Rv1 and normal prostatic epithelial cell line RWPE‐1. Surprisingly, our results showed that overexpression of SOX9 or KLF5 in 22Rv1 or RWPE‐1 cells led to varied outcomes. In the 22Rv1 cell line, the Wnt/β‐catenin pathway could be mildly activated, whereas in the RWPE‐1 cell line, it was dramatically activated. The downstream effector, the stemness marker KRT13, was dramatically upregulated in both cell lines (Figure 3L,M). CCK‐8 results showed that upregulation of SOX9 or KLF5 inhibited the growth of 22Rv1 cells but promoted the growth of RWPE‐1 cells (Figure S7G, Supporting Information). Immunocytochemistry results of RWPE‐1 clearly showed the nuclear translocation of β‐catenin after SOX9 or KLF5 activation (Figure 3N; Figure S7H, Supporting Information). The ChIP‐PCR and ChIP‐Seq results showed that CTNNB1 was the direct target of SOX9, KLF5, and ELF3 (Figure 3O,P). Taken together, these results reveal that after ADT, the residual cell type harbors a stem cell‐like state and may be associated with PCa recurrence.

### 2.4 Drafting of the Tumor‐Infiltrating Lymphocytes and Identification of the Effector CD8^+^T Cell Subset in the PCa Microenvironment

**Figure 4:**
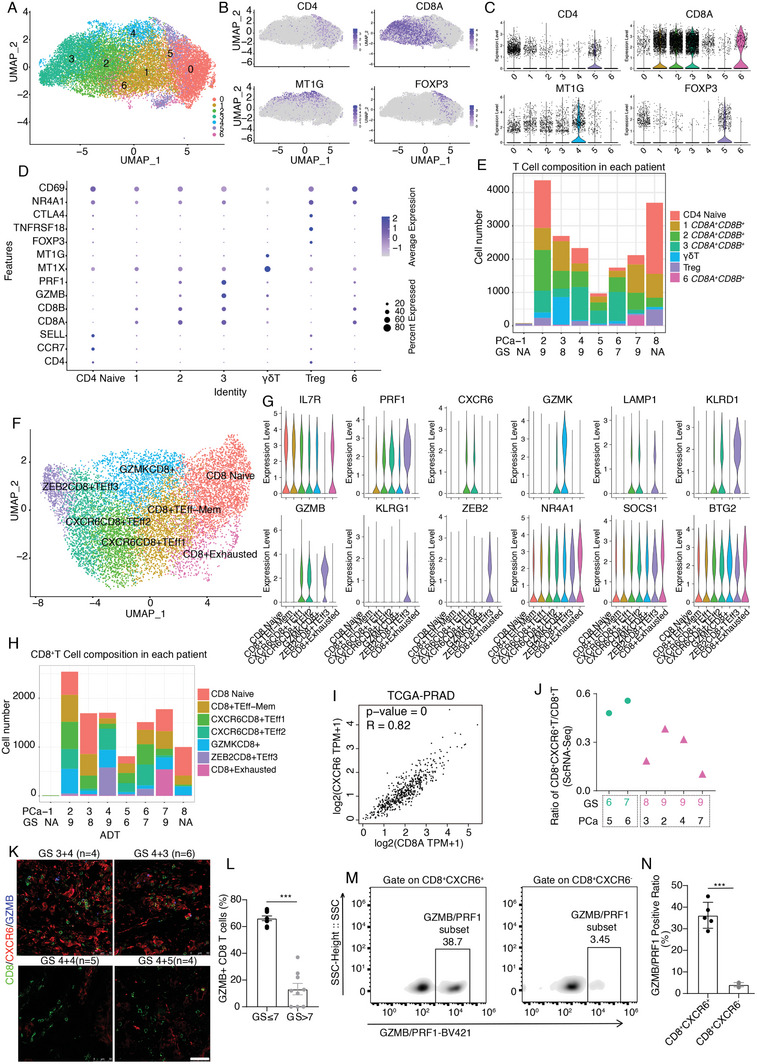
Effector CD8^+^T cell subset identification in the PCa microenvironment. A) Unsupervised clustering of T cells gave rise to 7 sub‐clusters. B–D) Comprehensive B) utilize feature plot, C) violin plot, and D) dot plot to illustrate the marker genes expression pattern in CD4T (*CD4*), CD8T (*CD8ACD8B*), γδT (*CD4‐CD8^−^MT1G^+^*) as well as regulatory T cells (*CD4* ^+^ *FOXP3*+). E) T Cell subset composition in each patient. The NE patient with the least T cell infiltration, and most infiltrating T cells are Tregs. F) Re‐cluster of CD8^+^T cells gave rise to 7 different subgroups of CD8^+^T cells. G) Violin plot illustrated the marker genes among the 7 CD8^+^T subgroups. The long‐lived effector CD8^+^T cells marked with *CXCR6* expression. H) NE patient (PCa‐1) with the least CD8^+^T lymphocyte infiltration, while the CRPC patient (PCa‐7) significantly enriched with the exhausted CD8^+^T (CD8^+^T Exhausted subtype labeled in pink) lymphocytes. In contrast, short‐term exposure to ADT therapy (PCa‐4) induced cell death co‐occurrence with activated CD8^+^T cells. I) CD8A and CXCR6 have a positive co‐expression correlation with in the TCGA‐PRAD cohort (Pearson correlation coefficient *R* = 0.82). J) scRNA‐Seq data suggested the ratio of CD8^+^CXCR6^+^ T cells in CD8^+^T cells reduced in the malignant PCa patients. Green dots indicated CD8^+^CXCR6^+^ T cells in GS low patients, pink triangles indicated CD8^+^CXCR6^+^ T cells in GS high patients. K,L) Immunofluorescence staining results suggested the number of *CD8^+^CXCR6^+^* T cells reduced in the malignant PCa patients. Scale bar: 50 µm. (*n* = 10 for GS≤7, *n* = 9 for GS>7). *, *p* < 0.05. **, *p* < 0.01, ***, *p* < 0.001. Mann–Whitney U‐test. M,N) CD*8^+^CXCR6^+^* T cells secreted more GZMB and PRF1 compared to CD8^+^CXCR6^−^ lymphocytes after stimulation. (*n* = 5 per group). ***, *p* < 0.001. Mann–Whitney U‐test.

Like many other solid tumors, PCa is considered poorly immunogenic or “immune desert,” lacking immune infiltration.^[^ ^19^ ^]^ However, our scRNA transcription results suggested that T cells ranked second among all cell types (Figure 1C). To further delineate the cellular composition and functional state of the infiltrating lymphocytes, we reanalyzed the T cell subsets and annotated the T cell subclusters with well‐established markers. Unsupervised clustering of T cells identified seven potential sub‐clusters (**Figure** **4**A). To better understand the features of the seven subclusters, the well‐established markers, *CD4* (CD4T*), CD8A* or *CD8B* (CD8T)*, FOXP3* (Regulatory T cells, Tregs), and γδT (CD3E^+^ CD4^−^CD8^−^ *MT1X^+^ MT1G^+^*) cells, were analyzed in all seven subclusters populations (Figure 4B–D).

Unexpectedly, we found that most infiltrating lymphocytes were CD8^+^ T lymphocytes (Figure 4B,E). CD8^+^T cells play a central role in antitumor immune responses by directly killing cancer cells.^[^ ^20^ ^]^ Therefore, we focused on this population in the present study. Seven different subgroups of CD8^+^T cells were characterized using different marker genes (Figure 4F,G; Figure S8A, Supporting Information). Our results showed that the number of functional CD8^+^T cells inversely correlated with GS. The patient with NEPC (PCa‐1), the most aggressive PCa phenotype, displayed the least CD8^+^ T‐lymphocyte infiltration (Figure 4H). In contrast, the patient with CRPC (PCa‐7) showed highly enriched exhausted CD8^+^ T lymphocytes (marked by *NR4A1* and *BTG2*). We further noticed that short‐term exposure to ADT (PCa‐4) induced cell death co‐occurring with activated CD8^+^T cells (marked with *PRF1^High^KLRD1^High^GZMB^High^ZEB2^+^KLRG1^+^IL7R^−^*); however, this subset belongs to short‐lived effector T cells. In addition, GZMK^+^CD8T, a hallmark of the aging immune population,^[^ ^21^ ^]^ occurred in all patients with prostate adenocarcinoma and the normal control (Figure 4H). Moreover, two long‐lived effector CD8^+^T subsets (marked by *IL7R, LAMP1* (CD107a), *and KLRD1*) co‐expressed *C‐X‐C Motif Chemokine Receptor 6(CXCR6)*. This encouraged us to determine whether long‐lived CD8^+^T cells co‐expressing CXCR6 were a general phenomenon in the PCa microenvironment. As expected, CD8A and CXCR6 were positively correlated in the TCGA‐PRAD cohort (Pearson correlation coefficient *R* = 0.82) (Figure 4I). More importantly, we found that CD8^+^T cells gradually lost their CXCR6 expression as PCa progressed (Figure 4J–L). These results indicated that CXCR6 expression is critical for CD8^+^T cell survival and cytotoxicity.

To test the differentiation process of these CD8^+^T cells in silico, we performed pseudo‐time analysis using Monocle 2. Pseudo‐time analysis showed that infiltrating CD8^+^ T lymphocytes could be subdivided into five states. State 1 was mainly composed of naïve CD8^+^ T cells, whereas the end‐point states (states 4 and 5) had the least number of naïve CD8^+^ T cells and were mainly composed of effector CD8^+^T subsets (Figure S8B–D, Supporting Information). A pseudo‐time heatmap revealed a continuous transition of CD8^+^T cells (Figure S8E, Supporting Information). We also found that naïve (state 1) to effector (states 4 and 5) switches were concurrent with the continuous activation of *CXCR6*, *PRF1*, and *TBX21* (Figure S8F, Supporting Information). Flow cytometry results using localized PCa samples without ADT showed that upon stimulation, CD8^+^CXCR6^+^ lymphocytes secreted more GZMB and PRF1 compared to CD8^+^CXCR6^−^ lymphocytes (Figure 4M,N), indicating that CD8^+^CXCR6^+^ lymphocytes can mediate target‐cell death by cytotoxic granule proteins. CXCR6 loss in CD8^+^ lymphocytes may, therefore, accelerate cancer progression.

### 2.5 TAM‐Macro‐2 is a PCa‐specific Tumor‐Associated Class of Macrophages and ADT Induces IL1B‐NLRP3 Macrophages with Potential Anticancer Activity

**Figure 5:**
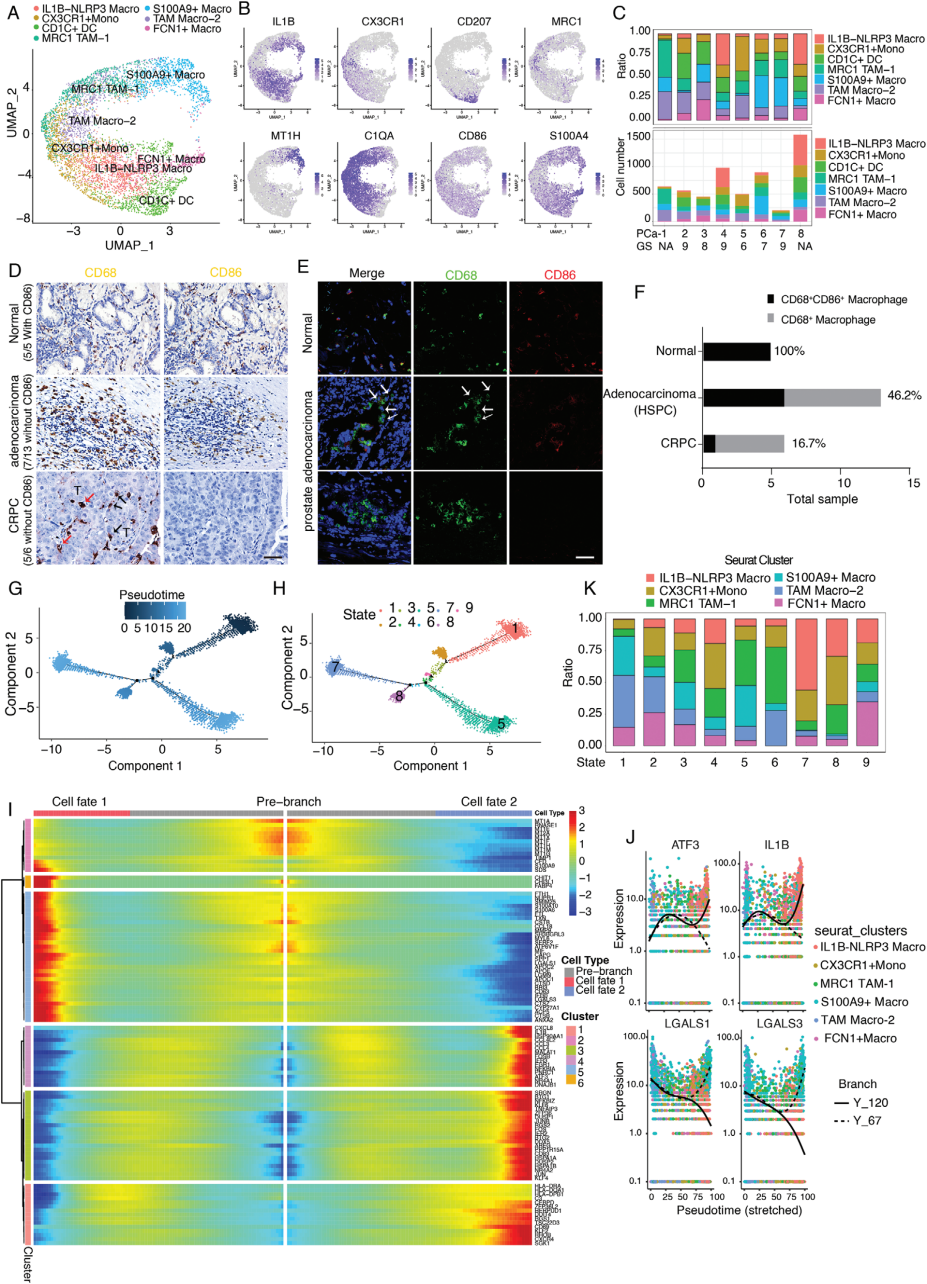
TAM‐Macro‐2 is a PCa‐specific tumor‐associated class of macrophages and ADT induces IL1B‐NLRP3 macrophages exhibiting potential anticancer activity. A) Reclustered myeloid cells were categorized into seven cell types by scRNA‐seq. B) Visualizing the marker genes expression pattern in a feature plot. C) Myeloid cell composition illustrated in ratio (upper panel) and absolute cell number (lower panel) in each patient. The TAM Macro‐2 loses the expression of CD86, almost exclusively found in cancer patients. D) IHC staining and E) immunofluorescence staining results suggested macrophage gradually lost the CD86 expression with tumor progression. Red arrows: macrophages infiltrated into the tumor region. Black arrows: macrophages surround the tumor. T: tumor. White arrows: macrophages with CD68 expression but lost CD86 expression. Scale bar: 50 µm. F) Stack histogram illustrated the ratio of CD68^+^CD86^+^ macrophages in CD68^+^ macrophages among the IHC samples. G,H) Pseudotime cell trajectories analysis using Monocle 2 to study the macrophage cellular dynamic processes visualization in pseudotime (G) or state (H). I) BEAM analysis in Monocle2 to find genes associated with branching in macrophage fate decisions. We plot the top 100 genes using the “plot_genes_branched_heatmap” function and “qval < 1e‐4″. J) Visualize the BEAM results using specific genes, such as *ATF3*, *IL1B*, *LGALS1*, and *LGALS3*, which are associated with cell fate decisions. Here we plot 2 representative genes highly expressed in cell fate2 (*ATF3* and *IL1B*), and 2 representative genes highly expressed in cell fate1 (*LGALS1* and *LGALS3*) as controls. K) Cellular composition in each state.

Consistent with previous reports,^[^ ^5^, ^13^ ^]^ myeloid cells comprised the second‐largest immune cell population in the PCa microenvironment. Reclustered myeloid cells, including monocyte, macrophage, and dendritic cells, were categorized into seven cell types by scRNA‐seq: IL1B‐NLRP3 Macro (*IL1B^High^NLRP3^High^*), CX3CR1^+^ Mono (*CX3CR1^High^CSF1R^High^*), CD1C^+^ dendritic cells (*CD14^−^CD1C^+^CD207^+^CLEC10A^+^*), tumor‐associated macrophages (MRC1‐TAM‐1, *CD163^High^SIRPA^High^MRC1^+^FOLR2* ^+^), S100A9^+^Macro (*S100A9^+^MT1H^+^*), TAM Macro‐2 (*C1QA^+^C1QB^+^C1QC^+^CD86^−^ CDKNA1^−^CCL3^−^*), and FCN1^+^ Macro 7(*FCN1^+^S100A4^High^IL1B^+^*) (**Figure** **5**A–C; Figure S9A, Supporting Information). Notably, IL1B‐NLRP3 and S100A9^+^ macrophages were enriched in PCa‐4 and PCa‐6 patients, respectively (Figure 5D). TAM Macro‐2 loses the expression of CD86, a potent co‐stimulatory signal necessary for T cell activation and survival, and is almost exclusively found in patients with cancer; therefore, we termed this subset TAM Macro‐2. This subset does not secrete T cell chemotactic factors such as *CCL3*, *CCL4*,^[^ ^22^ ^]^ or *CXCL8* (Figure 5D; Figure S9B,C, Supporting Information), demonstrating that this subset does not contribute to T cell trafficking regulation. Enrichment analysis results suggested that IL1B‐NLRP3 Macro regulated the inflammatory response and negatively regulated cell proliferation, suggesting its potential effects on anticancer activity (Figure S9D, Supporting Information). Compared to the normal prostate, the number of CD68^+^CD86^−^ macrophages increased in malignant tissues, as illustrated by immunohistochemical and immunofluorescence staining (Figure 5D,E). Interestingly, we found that TAM Macro‐2 was preferentially enriched in the interstitial region but also infiltrated the tumor region, indicating that tumor cells may hijack CD68^+^CD86^−^ to restrain the immune response and promote tumor growth (Figure 5D). In addition, enrichment analysis results indicated that TAM Macro‐2 functions in neutrophil degranulation, an emerging pathway that may facilitate cancer metastasis into different tissues, further suggesting that this subpopulation promotes cancer invasion (Figure S9E, Supporting Information). Pseudotime analysis of macrophage cells using Monocle2 reconstructed 2 different evolutionary trajectories (Figure 5G,H). This suggests that macrophages within the PCa microenvironment exhibit two major developmental and differentiation routes for cell fate decisions. We performed Branch Expression Analysis Modeling (BEAM) to identify the key genes responsible for defining branches within the macrophage pseudotime trajectory. We identified over 1000 significantly regulated genes (qval < 1e‐4, 1127 genes) during macrophage specification, resulting in six different gene expression modules along with macrophage cell fate specification (Figure 5I; Figure S10A, Supporting Information). ATF3 (Activating Transcription Factor 3) and IL1B (Interleukin 1 Beta), both canonical markers of activated macrophages, were highly expressed in cell fate 2 and were involved in pro‐inflammatory cytokine production (Figure 5I,J). Their high expression in cell fate 2 indicates a potential pro‐inflammatory phenotype or activation state in these cells. In contrast, LGALS1 (Galectin‐1) and LGALS3 (Galectin‐3), both known to influence macrophage polarization toward an immunosuppressive state, demonstrate elevated expression levels in cell fate 1. This suggests a propensity toward an immunosuppressive or anti‐inflammatory phenotype within the cells assigned to fate 1 (Figure 5I,J).

We also explored their cellular composition in each “State” along the pseudotime. State 5 mainly consisted of MRC1 TAM‐1 and S100A9+ Macro subsets, while state 7/8 was mainly composed of IL1B‐NLRP3 and CX3CR1^+^ Mono subsets (Figure 5K). Moreover, we visualized the gene modules that corresponded to our six subsets, such as *C1QC*, *CX3CR1*, *CCL3*, *IL1B*, *S100A9*, *FCN1*, and *MRC1* with pseudotime heatmap. These genes exhibited both continuous and lineage‐dependent expression patterns (Figure S10B, Supporting Information). We further explored the chemokines (*CCL3* and *IL1B)* and cell surface markers with signal regulatory protein (*CD86, MRC1*, and *SIRPA)* expression patterns in different states. Consistent with our results, chemokines that regulate immune cell trafficking dramatically increased in state 7, while *MRC1* and *SIRPA*, both well‐known immunosuppressive markers, dramatically decreased in state 7 (Figure S10C,D, Supporting Information). Taken together, we identified common and distinct gene regulation patterns during macrophage specification.

Given the complex role of tumor‐infiltrating macrophages in the TME, we investigated which subset of macrophages functions as defenders or betrayers to contribute to the anticancer response or promote cancer cell growth and metastasis. Characterization of cell‐cell communication using CellChat^[^ ^23^ ^]^ indicated sophisticated cell‐cell interactions in the PCa microenvironment (Figure S11A,B, Supporting Information). Our results showed that the MHC‐I signaling pathway is a universal crosstalk among the myeloid‐epithelia‐T cell subsets, albeit the interaction strength differs among these cells (Figure S11B,C, Supporting Information).

Among them, the CD8^+^CXCR6^+^ T cell subset with a noticeable MHC‐I signaling pathway interaction number and strength with other cell types indicated its central role in the antitumor immune response (Figure S11C, Supporting Information). In addition, we found that macrophage subsets communicate with CD8^+^CXCR6^+^ T cells via the CD99, ADGRE5, APP, and CLEC signaling pathways (Figure S11D–G, Supporting Information). It is well known that C‐type lectins are implicated in a diverse range of physiological functions in mammalian immunity and homeostasis. Our results showed that the CLEC signaling pathway‐CLEC2B/C_KLRB1 interaction (Figure S12A,B, Supporting Information) might be the prominent pathway that shapes CD8^+^CXCR6^+^T in anticancer responses.

In addition, we investigated the spatial co‐location of T cells with other cell types (Figure S11H, Supporting Information). Deconvolution results suggested that compared to epithelia, immune cells are closer to each other, indicating activated cell communication between the immune cells to promote or restrain the immune response.

### 2.6 Conflicting functions of Two Distinct States of Intratumoral Neutrophils

**Figure 6:**
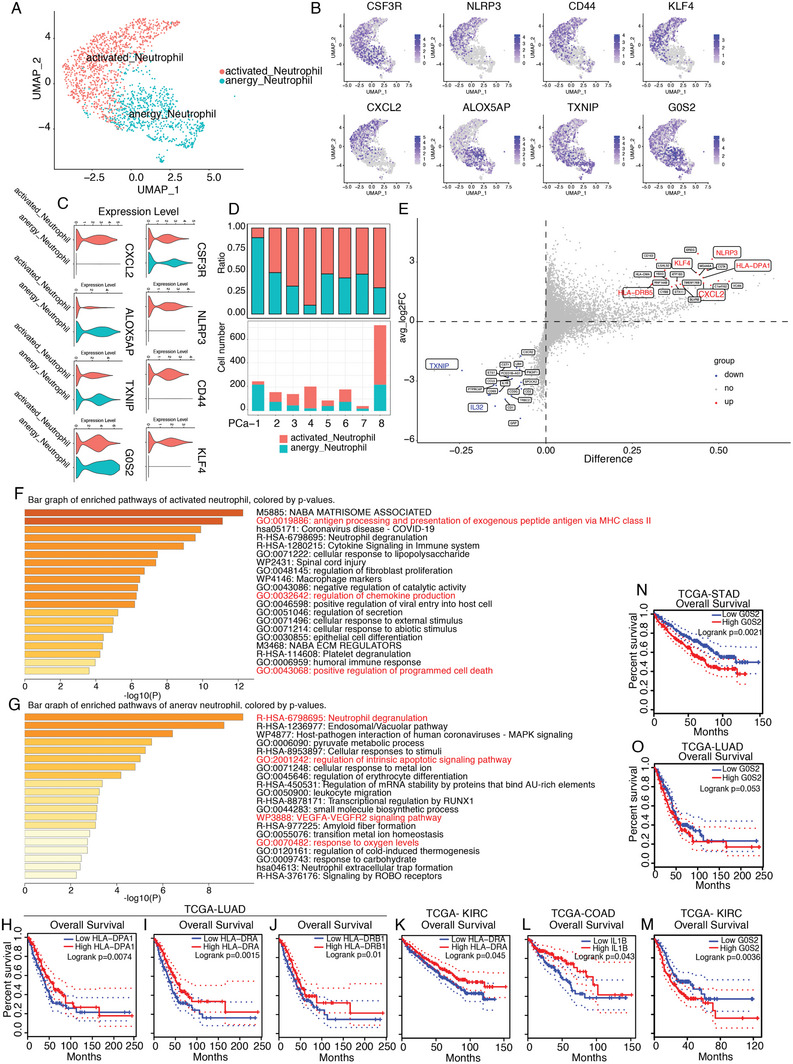
Intratumoral neutrophils display conflicting functions. A) Reclustering of intratumoral neutrophils was categorized into “activated neutrophils” and “anergy neutrophils”. B,C) Visualizing the marker genes expression pattern in B) feature plot or C) expression level in violin plot. Neutrophil activation markers‐ *NLPR3* and *CD44*, expressed highly in the “activated neutrophils” subset. D) Neutrophils composition illustrated in ratio (upper panel) and absolute cell number (lower panel) in each patient. The ratio of “anergy neutrophils” in NE patients is significantly higher than the other patients. E) Volcano plot to show the differentially expressed genes between the subsets of neutrophils. F,G) Pathway enrichment analysis suggested “activated neutrophils” play an antitumor role while the “anergy neutrophils” display conflicting functions in PCa. All gene ontology (GO) analyses were performed with the online tool–Metascape (http://metascape.org).^[^ ^53^ ^]^ H–J). Higher HLA‐DPA1, HLA‐DRA, and HLA‐DRB1 expression levels indicated a good prognosis in LUAD. K,L) Higher HLA‐DRA and IL1B expression levels predicted a good overall survival of KIRC and COAD, respectively. M–O) G0S2 is linked to poor prognosis in several tumor types, including M) KIRC, N) STAD, and O) LUAD.

Neutrophils, the most abundant myeloid cells in human blood, infiltrate the PCa microenvironment at a lower level (Figure 1B,C); however, their functions in the PCa microenvironment remain unclear. One subset of infiltrating neutrophils highly expressed *NLPR3*, *CD44*, and *KLF4*, whereas the other subset expressed different genes, such as *ALOX5AP* and *TXNIP*. Given the fact that NLPR3 is the well‐established marker of neutrophil activation,^[^ ^24^ ^]^ we distinguished the neutrophils subset as “activated neutrophils” or “anergy neutrophils” (**Figure** **6**A–C). Interestingly, both activated and anergy subsets infiltrated all patients. However, the ratio of anergic neutrophils increased markedly in patients with advanced‐stage PCa‐1 (Figure 6D). We also observed that the ratio of activated neutrophils markedly increased after ADT (Figure 6D).

The transcriptional profile differences between “activated neutrophils” and “anergy neutrophils” were further investigated. We found that, compared to the anergy subset, the activated subset expressed more *HLA‐DPA1*, *HLA‐DRB5*, and *CXCL2* (Figure 6E), indicating that the activated subset may possess antigen presentation via MHC Class II molecules as well as immune cell recruitment. Pathway enrichment analysis suggested that the genes expressed in activated neutrophils mainly participated in antigen processing, presentation of exogenous peptide antigens via MHC class II molecules, and regulation of chemokine production (Figure 6F). The upregulated genes in anergic neutrophils enriched pathways including neutrophil degranulation, regulation of the intrinsic apoptotic signaling pathway, the VEGF‐VEGFR2 pathway, response to oxygen levels, and neutrophil extracellular trap formation (Figure 6G). It is well known that the VEGF‐VEGFR2 pathway promotes tumorigenesis, and the neutrophil extracellular trap formation hinders cytotoxic CD8^+^T cell‐induced antitumor immunity.^[^ ^25^ ^]^ These results suggest that anergic neutrophils play a tumor‐promoting role.

Furthermore, we assessed whether the intratumoral neutrophil status was associated with cancer progression. The top genes of activated neutrophils, namely HLA‐DPA1, HLA‐DRA, and HLA‐DRB1, were found to be associated with a good prognosis in lung adenocarcinoma (LUAD). In addition, higher HLA‐DRA or IL1B expression levels also predicted good overall survival in kidney renal clear cell carcinoma (KIRC) and colorectal adenocarcinoma (COAD), respectively (Figure 6H–L). These results indicate that activated neutrophils may play an antitumor role. In contrast, G0S2, a highly expressed gene in anergic neutrophils, was associated with poor prognosis in several tumor types, including KIRC, stomach adenocarcinoma (STAD), and LUAD (Figure 6M–O). These results indicate that activated neutrophils may play an antitumor role, while anergic neutrophils alter their role from protective to potentially tumor‐promoting in PCa.

### 2.7 Role of Heterogeneity of Fibroblast Subpopulations

**Figure 7:**
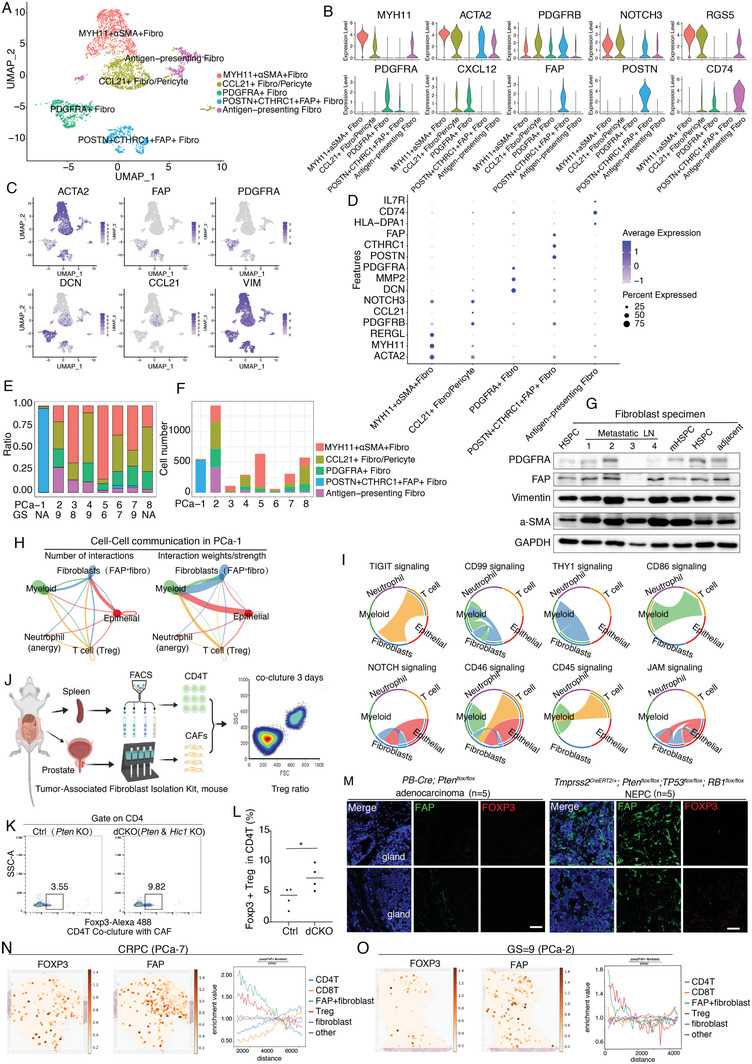
Characterize the heterogeneity of fibroblast subpopulations. A) scRNA‐seq of PCa fibroblasts revealed five distinct cell types based on their transcriptional profiles. B–D) Comprehensive utilized B) violin plot, C) feature plot, and D) dot plot to illustrate their marker genes expression pattern among the 5 distinct fibroblast subpopulations. E,F) Fibroblasts composition illustrated in ratio E) and absolute cell number F) in each patient. Both the ratio and absolute cell number of *POSTN^+^CTHRC1^+^FAP^+^* Fibro in NE patients are significantly higher than in the other patients. G) Comprehensively utilized PDGFRA, FAP, Vimentin, and ACTA2 to discriminate the subset of fibroblasts in the PCa microenvironment. Western blot analysis of cell lysates was performed using antibodies against PDGFRα, FAP, Vimentin, α‐SMA, and GAPDH. H) Cell‐cell communication results showed fibroblasts mainly interact with myeloid cells and epithelial cells, followed by T cells. I) Visualizing the cell‐cell communication by CellChat revealed the major signaling pathways in PCa‐1. J) Illustration of the CAFs co‐culture with CD4T cells. Figures created with BioRender (https://biorender.com/). K,L) Flow cytometry showed CAFs isolated from *dCKO* mice induce more Treg differentiation compared with *Ctrl* mice. (*n* = 4 per group). *, *p* < 0.05. Mann–Whitney U‐test. M) Immunofluorescence staining results suggested the NEPC mouse prostate with abundant FAP^+^ fibroblasts and their space location close to FOXP3^+^ Treg in the prostate. The control mice with little or no FAP^+^ fibroblasts. Scale bar: 50 µm. N,O) In situ analysis of FOXP3^+^ regulatory T cells and FAP^+^ fibroblasts in the malignant PCa patients. The line plot indicated the co‐occurrence enrichment of non‐epithelial cell types surrounding FAP^+^ fibroblasts.

Fibroblasts can serve as positive regulators of tumor progression by promoting angiogenesis, regulating tumor immunity, and modulating chemoresistance.^[^ ^26^ ^]^ It is now widely accepted that CAFs are a heterogeneous population with distinct functions. Our scRNA‐seq data from PCa fibroblasts revealed five distinct cell types based on the expression of a unique repertoire of collagen and other extracellular matrices (ECM) molecules (**Figure** **7**A–D; Figure S13A, Supporting Information). Further characterization showed that one of the subsets highly expressed several genes associated with myogenesis (including *ACTA2, MYH11 and RERGL*, which we termed *MYH11^+^a‐SMA^+^* Fibro) in one subpopulation, whereas another subset showed strong expression of genes with similar characteristics to *MYH11^+^a‐SMA^+^* Fibro but marked by *PDGFRB, CCL21, NOTCH3*, and *RGS5* expression, suggesting that this subset may be pericytes or of a potential pericyte origin (Figure 7B–D). Intriguingly, we found a subset of uniquely expressed *PDGFRA* and highly expressed ECM‐related genes, *MMP2*, *DCN*, and *CXCL12*, which we termed *PDGFRA^+^* Fibro. Notably, a separate subset of fibroblast (which we termed *POSTN^+^CTHRC1^+^FAP^+^* Fibro) highly expressed fibroblast‐activation protein‐α (FAP), periostin (POSTN), MMP11, and collagen triple helix repeat containing 1 (CTHRC1) (Figure 7D; Figure S13A, Supporting Information), suggesting that this subset was in an activation state and predominant in patients with NEPC (Figure 7E,F). Antigen‐presenting fibroblasts constitute another subset marked by *CD74*, *HLA‐DPA1*, and *IL7R* expression, and the ratio of this subset increased with increasing GS (Figure 7E). Pathway analysis showed genes in *MYH11^+^a‐SMA^+^* Fibro mainly associated with smooth muscle contraction and vascular smooth muscle contraction, while genes in *CCL21^+^* Fibro/Pericytes were mainly associated with response to interferon‐gamma and negative regulation of immune system process (Figure S13B,C, Supporting Information).

The extracellular matrix organization (ECM) pathway was the most common and top‐ranked pathway in both *PDGFRA^+^* Fibro and *POSTN^+^CTHRC1^+^FAP^+^* Fibro subsets. In addition to the ECM pathway, the enriched pathways in *POSTN^+^CTHRC1^+^FAP^+^* Fibro subset included collagen metabolic processes and ECM‐receptor interactions (Figure S13D,E, Supporting Information). These results indicate that fibroblasts with FAP and/or PDGFRA activation may be the result of tumor microenvironment remodeling under different circumstances. Indeed, the comprehensive application of PDGFRA, FAP, Vimentin, and ACTA2 for discerning fibroblast subsets in the PCa microenvironment successfully distinguished fibroblasts not only from the adjacent tissue but also from HSPC patients exhibiting lower expression levels of PDGFRA, FAP, Vimentin, and α‐SMA. In contrast, fibroblasts in the metastatic tissues or mHSPC samples had robust PDGFRA, FAP, Vimentin, and a‐SMA expression (Figure 7G).

In the NEPC sample, *POSTN^+^CTHRC1^+^FAP^+^* Fibro subset was predominant, and most infiltrating T cells were Treg cells (Figure 4E), suggesting that *POSTN^+^CTHRC1^+^FAP^+^* Fibro may be associated with immunosuppressive functions, including recruitment of *CD4^+^CD25^+^* T cells and/or survival and differentiation of *FOXP3^+^* Tregs. The cell‐cell communication results showed that fibroblasts mainly interacted with myeloid and epithelial cells, followed by T cells (Figure 7H). Moreover, TIGIT signaling was the exclusive pathway found only in Fibroblast‐T cell communication, suggesting that *POSTN^+^CTHRC1^+^FAP^+^* Fibro may promote Treg survival and/or differentiation (Figure 7I). To verify our hypothesis, we isolated CAFs from prostate tissue and extracted CD4^+^T cells from the spleen to test whether CAFs could promote CD4^+^T cell differentiation (Figure 7J). Using our established PCa mouse model, we found that CAFs isolated from *dCKO* (*PB‐Cre; Pten^flox/flox^; Hic1^flox/flox^*) mice^[^ ^27^ ^]^ induced greater Treg differentiation than those isolated from *Ctrl* (*PB‐Cre; Pten^flox/flox^*) mice (Figure 7K–L; Figure S13F, Supporting Information). To visualize and study the spatial distribution of FAP^+^ fibroblasts and FOXP3^+^ regulatory T cells, we used immunofluorescence staining of mouse tissues and immunohistochemistry (IHC) of human prostate samples, respectively. Both results revealed the physical proximity of FAP^+^ fibroblasts and FOXP3^+^ Treg cells in the NEPC mouse model^[^ ^28^ ^]^ and in human NEPC tissues (Figure 7M; Figure S14A,B, Supporting Information), supporting the direct interaction between them. In addition, spatial transcription showed the proximity of FAP^+^Fibro to T cells, suggesting that FAP^+^ fibroblasts may interact with FOXP3^+^Treg directly (Figure 7N,O).

scRNA‐Seq data suggested that the *POSTN^+^CTHRC1^+^FAP^+^* Fibro subset had the most abundant TGF‐β expression compared to the other fibroblast subsets (Figure S13G, Supporting Information). Moreover, the TGF‐β pathway ranks first when CellChatDB^[^ ^23^ ^]^ “Secreted Signaling” was used for cell‐cell communication analysis. It is well‐known that TGF‐β is a potent inducer of Treg differentiation, indicating that the FAP^+^ Fibro might induce Treg differentiation via TGF‐β signaling (Figure S15A–C, Supporting Information).

To further investigate the clinical implications of FAP^+^ fibroblasts in promoting PCa progression, we compared overall survival (OS), disease‐free survival (DFS), and progression‐free interval (PFI) between patients with different levels of FAP^+^ fibroblasts. We use the top 10 genes expressed (*POSTN, CTHRC1, GRP, LOXL2, LOX, COL11A1, FAP, DUXAP8, TNFAIP6*, and *COL5A1*) in FAP^+^ fibroblasts as the gene signatures as well as the *FAP* itself to reassess the relationship between the gene expression level with the disease prognosis. Although the OS did not show a significant difference, DFS using the top 10 gene signatures was more significant than that using only a single FAP gene (*P* = 0.004 vs. *P* = 0.05; Figure S16A–D, Supporting Information). Moreover, lower FAP levels predicted a longer PFI (*P* = 0.005902) in the TCGA‐PRAD cohort (Figure S16E, Supporting Information), indicating that FAP^+^ fibroblasts may contribute to PCa progression by creating a niche to promote chemotherapy or radiotherapy resistance. In summary, we reclassified the fibroblast subset and identified a FAP^+^ fibroblast subset as CAFs with an immune regulatory function in the PCa microenvironment, which could create an immunosuppressive microenvironment to promote tumor progression.

## 3 Discussion

Tumor progression is profoundly influenced by interactions between cancer cells and their environment, which ultimately determine whether the primary tumor is eradicated, metastasizes, or establishes dormant micrometastases.^[^ ^29^ ^]^ It is believed that the TME is not just a silent bystander but rather an active promoter of cancer progression. In this study, we generated 77 661 single‐cell transcriptomes from seven PCa patients paired with one normal sample, focusing on the cellular phenotypes associated with the PCa ecosystem. We comprehensively utilized machine learning and computational intelligence to identify the cellular diversity of the prostate and cell‐cell communication in situ. Here, we explored the roles of epithelial heterogeneity and lineage plasticity in PCa progression. Moreover, we characterized tumor‐infiltrating immune cells and stromal cells not only in terms of cellular composition but also in their functional state.

A recent study revealed that a subset of patients with PCa (3/21, referred to therein as “converters”) progressed after enzalutamide treatment possibly due to enza‐induced lineage plasticity. Master regulator analysis revealed that E2F1 was the top transcription factor predicted to be activated in the “converters”.^[^ ^2a^ ^]^ Herein, we present a different approach. We found that *SOX9*, the core transcription factor involved in the initiation of prostate organogenesis and carcinogenesis, acts as a driver that promotes the transition of club cells into a stem cell‐like state. Previous studies have shown that transient SOX9 expression facilitates ADT resistance in PCa.^[^ ^30^ ^]^ Another study reported that SOX9 drives WNT/β‐catenin signaling activation in PCa.^[^ ^31^ ^]^ These results, together with our findings, suggest that a subgroup of patients with PCa would benefit from WNT‐targeted therapy alone or in combination with ADT.

In addition, one study showed that PCa epithelium‐upregulated NPY could antagonize apoptosis, increase motility, and cause energetic metabolic pathway changes.^[^ ^32^ ^]^ Here, we found that NPY was upregulated in low‐grade cancer cells and normal‐like epithelial subpopulations, indicating that NPY may serve as a driver or indicator of PCa initiation. We also found that another Kallikrein, *KLK12*, a sensitive marker of CRPC, may be an alternative to *KLK3*.

Advanced age is one of the strongest risk factors for PCa. Here, we found that GZMK^+^CD8^+^T cells, an indicator of immunosenescence, were present in all eight patients. Senescent T cells exhibit abnormal phenotypes, including the downregulation of the co‐stimulatory molecule CD28 and the upregulation of CD57, Tim‐3, TIGIT, and CTLA‐4, which are closely associated with malignant tumors.^[^ ^33^ ^]^ Functional experiments demonstrated that these *GzmK^+^GzmB^+^* CD8 T cells are major cytokine producers with low cytotoxic potential,^[^ ^34^ ^]^ suggesting that this subset may be a regulator of the immune response instead of effector cells. Using scRNA sequencing, flow cytometry, and immunofluorescence staining, we identified effector T cells in PCa with extensively enhanced *CXCR6* expression, a critical receptor that receives signals for cytotoxic T cells survival in the tumor microenvironment.^[^ ^35^ ^]^ The number of *CD8^+^CXCR6^+^* T subsets is markedly reduced in patients with malignant PCa. Besides *GZMK^+^CD8^+^* T cells, age‐modified tissue‐specific macrophages and neutrophils may cause chronic low‐grade inflammation, which is related to macrophage‐mediated immunosuppressive disorders, leading to the development of cancer.^[^ ^36^ ^]^ We found that IL1B‐NLRP3 macrophages and activated neutrophils were the predominant subtypes of macrophages and neutrophils, respectively, in normal controls. *MRC1^+^* TAM‐1 cells, as well as anergy neutrophil subpopulations, are also subsets with a relatively high abundance that cannot be ignored. These cells may create an immunosuppressive environment that favors tumor escape.

Notably, we identified a new macrophage subset, which we referred to as “TAM Macro‐2,” characterized by the absence of *CD86* and *CDKN1A* expression. *CD86* is a canonical marker of “M1” macrophages. However, various studies revealed that TAMs, often resembling the polarized “M2” macrophages, also expressed the “M1” macrophage marker *CD86*. Given that CD86, as a co‐stimulatory molecule, interacts with CD28 for T‐cell activation and survival,^[^ ^37^ ^]^ the loss of CD86 expression in TAM Macro‐2 may result from a cancer cell‐induced program in the PCa microenvironment. It has been reported that clear cell renal cell carcinoma (ccRCC) hijacks macrophage‐produced complement C1q to promote tumor growth.^[^ ^38^ ^]^ Here, we found that “TAM Macro‐2” subsets surrounded the tumor region but also infiltrated into tumor, indicating that PCa cells may hijack the immune system to facilitate metastases in a similar way. Furthermore, we found that SIRPA (the “don't eat me” molecular CD47 ligand^[^ ^39^ ^]^) was overactivated in MRC1^+^TAMs and expressed on most of the tumor‐infiltrating macrophages, indicating that macrophages are important mediators of tumor immunosurveillance in the PCa microenvironment.

Intriguingly, the high tumor‐associated neutrophil (TAN) populations in the myeloid cell‐enriched subtype are associated with an unfavorable prognosis.^[^ ^40^ ^]^ Autologous *CD8^+^* T cells co‐cultured with human TANs (marked increase in *CD274*) exhibited lower proliferation properties as well as lower levels of cytotoxic granule proteins, whereas other *CCL4^+^* TAN subsets expressed high levels of the chemokine genes *CCL3* and *CCL4*, which could recruit macrophages,^[^ ^40^ ^]^ demonstrating that they have different functions. Here, we identified two subpopulations of neutrophils in the PCa microenvironment, marked with a high expression of *ALOX5AP* or *CD44*. ALOX5AP predicts poor prognosis by enhancing M2 macrophage polarization and immunosuppression in the serous ovarian cancer microenvironment,^[^ ^41^ ^]^ whereas ligation of CD44 triggers the lytic function of cytotoxic T cells and enhances natural killer cell activity.^[^ ^42^ ^]^ Taken together, these results demonstrate that tumor‐infiltrating cells exhibit distant functions in PCa.

Activated fibroblasts have been identified as CAFs that play a central role in multiple cancers, including PCa. CAFs are integral components of the tumor microenvironment. CAFs can facilitate tumor proliferation, invasion, and metastasis and are often associated with poor prognosis.^[^ ^43^ ^]^ Previously, we identified two distinct fibroblast clusters in the PCa microenvironment.^[^ ^13^ ^]^ We have also reported that CAFs crosstalk with tumor cells to promote breast cancer^[^ ^44^ ^]^ and PCa progression.^[^ ^27^ ^]^ A recent study revealed the conservation of three supercluster CAF phenotypes across species and tissues of origin.^[^ ^45^ ^]^ In line with this report, our data showed that the transcription profiles of CCL21^+^Fibro/Pericytes and antigen‐presenting fibroblast subclusters closely aligned with the immunomodulatory (IM) fibroblasts, and *PDGFRA^+^* and *POSTN^+^CTHRC1^+^FAP^+^* Fibro subsets could be referred to as mechanoresponsive (MR) fibroblasts. Intriguingly, the transcriptome of *MYH11^+^a‐SMA^+^* Fibro subset does not resemble that of the steady state‐like (SSL) fibroblasts. Considering that *MYH11^+^a‐SMA^+^* fibroblasts were mainly contributed by the PCa‐5 patient (GS:3+4), we speculated that this subset should be the SSL fibroblasts. These findings suggest that CAFs in relatively lower abundance in the PCa microenvironment also showed strong evolutionarily conserved phenotypes in the form of three transcriptionally delineated superclusters and modulated diverse functional elements, such as creating an immunosuppressive environment that affects tumor progression.

PCa is considered a poorly immunogenic tumor, sometimes referred to as an “immune desert,” that lacks immune cell infiltration. Our scRNA transcription results suggested that T cells and macrophages ranked second and third in all cell types, respectively. Our results may boost a reassessment of long view of PCa as an “immune desert”. For patients with abundant immune cell infiltration, immunotherapy may be an alternative option for precision medicine.

We found that club cells with self‐renewal capabilities and epithelial differentiation potential may constitute another source of PCa recurrence. In addition, we found that fibroblasts can create an immunosuppressive environment in malignant tumors. Considering the critical role of Tregs in tumor immune escape and the fibroblast‐derived extracellular matrix as a barrier to prevent drug penetration, patients suffering from cancer may benefit from the deprivation of fibroblast treatment.

In summary, we present a comprehensive investigation of PCa single‐cell gene expression and paired spatial transcriptomics in conjunction with bulk chromatin accessibility during PCa progression. Our data revealed epithelial heterogeneity and identified potential subtypes that might account for PCa recurrence. We explored the temporal and spatial dynamics of epithelia, immune cell biology, and their functional transition with tumor progression. Further exploration of the impact of epithelial–immune cell/stromal cell communication and delineation of the key regulatory pathways determining disease progression would offer new opportunities to better understand PCa biology and propose translational research paths for treating PCa.

## 4 Experimental Section

### Ethics Declarations—Ethics Approval and Consent to Participate

Prostate tissues were obtained from patients treated at the Fudan University Shanghai Cancer Center under Institutional Review Board‐approved clinical protocol 050432‐4‐2108*. Detailed patient information is provided in Table S1 in Supporting Information. All patients provided written informed consent.

### scRNA‐Seq Library Construction and Sequencing

Prostates were harvested and minced into small pieces (≈1 mm^3^ in size) on ice, then incubated in 0.2% Collagenase II (Gibco, #17101‐015) with 2.5U mL^−1^ Dispase II (Sigma, #D4693) (9:1 in volume) for 45 min in 37 °C water bath, pipetted every 5 min. The cell suspension was then filtered through a 40 µm cell strainer for single‐cell RNA‐Seq. The concentration of cell suspension was adjusted to 800–1100 cells per µl. Cells were loaded between 10 000 and 18 000 cells per chip position using the Chromium Next GEM Single Cell 5' Reagent Kits v2 (10x Genomics, Dual Index). Single‐cell gene expression libraries were generated according to the manufacturer's instructions, and single‐cell expression sequencing was run on a NovaSeq 6000 (Novogene). The 10x scRNA‐seq data were preprocessed using Cell Ranger software (6.0.1). We used the ‘mkfastq’, ‘count’, and ‘aggr’ commands to process the 10x scRNA‐seq output into one cell by gene expression count matrix using default parameters. Cells expressing <100 and >7000 genes were also removed from further analysis. In addition, cells with a high (≥0.15) mitochondrial genome transcript ratio were removed.

Alignment of sequencing data CellRanger toolkit v2.1.0 was used to process the raw sequencing data. Reads were aligned to the GRCh38/hg38 genome build. UMI count was quantified for each cell and the UMI matrices of all samples were filtered using default parameters. The filtered UMI matrices consisted total of 83 874 single cells.

### Cell Clustering and Annotation

UMI counts were log‐transformed and normalized by Seurat suit, as “Normalized expression value” (Expr.) used throughout the current study. PCA and cell graph‐based clustering were performed using the batch effect‐corrected matrices. After correcting the batch effects, there were 77 661 cells left, and annotated in agreement were included for downstream analysis.

### Data Merging and Filtering

Subsequent data analysis was performed using RStudio (v1.1.456, RStudio, Inc.) with R (v4.1.0, R Core Team, 2021). The majority of the analyses, including UMI log normalization, cell selection, principal component analysis (PCA), dimensionality reduction, cell clustering, and annotation, were all performed using Seurat suite (v3).^[^ ^46^ ^]^

### Pesdotime Analysis

We utilized Monocle (packageVersion = ‘2.22.0’) to order cells in pseudotime based on their transcriptomic similarity. For epithelial cell pseudotime analysis, the new package Monocle 3 was further employed (http://cole‐trapnell‐lab.github.io/monocle‐release/) to validate the robustness of trajectory analysis results. To find genes differentially expressed across the branch point in the trajectory, Monocle's internal BEAM analysis was used and genes with qval < 1e‐4 were selected. Gene expression patterns were plotted with plot_genes_branched_heatmap and plot_multiple_branches_pseudotime.

### Spatial Transcriptomics

Fresh prostates were dissected and storage in the tissue storage solution (Miltenyi Biotec; 130‐100‐008) for spatial transactional sequencing. Visium Spatial Gene Expression Slide and Reagent Kit (10X Genomics, 1000187) was sued to explore the gene expression within the tissue context.

For spatial transcriptome analysis, machine learning XGBoost algorithm (https://github.com/tqchen/xgboost) and computational intelligence/TensorFlow 2 (https://tensorflow.google.cn/) were comprehensively utilized to identify the cellular diversity of prostate in situ.

### Bulk Assay for Transposase‐Accessible Chromatin with High‐Throughput Sequencing (ATAC‐seq)

Nuclei suspensions were incubated in a Transposition Mix that includes a Transposase. The Transposase enters the nuclei and preferentially fragments the DNA in open regions of the chromatin. Simultaneously, adapter sequences were added to the ends of the DNA fragments. Incubate the transposition reaction at 37 °C for 30 min. Immediately following transposition, the products were purified using a QIAGEN minielute kit, amplified as described before,^[^ ^47^ ^]^ and sequenced using Illumina Novaseq 6000 (Novogene) by Gene Denovo Biotechnology Co. (Guangzhou, China). Bowtie2^[^ ^48^ ^]^ (version 2.2.8) with the parameters ″–X 2000″ was used to align the clean reads from each sample against the reference genome, and reads aligned to the mitochondria or chloroplasts were filtered. For all data files duplicates were removed using Picard. All reads aligning to the + strand were offset by +4 bps, and all reads aligning to the – strand were offset −5 bps. Shifted, concordantly aligned paired mates were used for peak calling by MACS (version 2.1.2)^[^ ^49^ ^]^ with parameters “–nomodel –shift ‐100 –extsize 200 ‐B ‐q 0.05”. MACS was a computational method that was designed to identify read‐enriched regions from sequencing data. Dynamic Possion Distribution was used to calculate *p*‐value of the specific region based on the unique mapped reads. The region would be defined as a peak when *q*‐value<0.05.

### Cell Lines

The human PCa cell lines 22Rv1 and human normal prostate epithelial cell line RWPE‐1 were obtained from the American Type Culture Collection (ATCC) and cultured according to standard protocols from the ATCC website. All cell lines were cultured in 1% penicillin/streptomycin (Gibco; 15140122) media at 37 °C and under 5% CO_2_.

### Mouse Strains

Double conditional knockout mice were generated by crossing *Hic1^flox/flox^* and *Pten^flox/flox^* mice with *PB‐Cre* mice in which Cre recombinase expression was driven by prostate‐specific rat probasin promoter (Pb) as described previously.^[^ ^27^ ^]^ The Cre‐positive; *Hic1^flox/flox^*; *Pten^flox/flox^* mice were designated as *dCKO*, which were identified as the test group; littermates, Cre‐positive; *Pten^flox/flox^* mice, were designated as the Pten^−/−^ control group (*Ctrl*). All mice were maintained under specific pathogen‐free (SPF) conditions with 12 h light/12 h dark cycles and temperature and humidity set points of 20–25 °C and 30–70%, respectively. All animal experiments were performed in accordance with protocols approved by the Institutional Animal Care and Use Committee of Fudan University Shanghai Cancer Center (Protocol number: FUSCC‐IACUC‐S2022‐0219).

### CAFs and CD4T Cells Enrichment

Mouse tumor‐associated fibroblasts (CAFs) were purified from spontaneous prostate cancer model (*dCKO* or *Ctrl* mice) using the Tumor‐Associated Fibroblast Isolation Kit, mouse (Miltenyi Biotec, Cat No. 130‐116‐474) according to the manufacturer's protocol. Briefly, mice were sacrificed and prostates or spleens were collected. Prostates were harvested and minced into small pieces (≈1 mm^3^ in size) on ice, then incubated in 0.2% Collagenase II (Gibco, #17101‐015) with 2.5U mL^−1^ Dispase II (Sigma, #D4693) (9:1 in volume) for 45 min in 37 °C water bath, pipetted every 5 min. Cell suspension was then filtered through a 70 µm cell strainer for CAFs enrichment. For pre‐enrichment an LD Column (Miltenyi Biotec; 130‐042‐901) and a QuadroMACS Separator (Miltenyi Biotec; 130‐090‐976) were used, subsequent isolation was performed on a MS Column (Miltenyi Biotec; 130‐042‐201) and a MiniMACS Separator (Miltenyi Biotec;130‐042‐303).

CD4T cells were isolated from the spleens and enriched by flow cytometry (MoFlo XDP, Beckman Coulter) with the PE anti‐mouse CD4 antibody (Cat. No. 100408; Biolegend). Red blood cells were eliminated with red blood cell lysis solution (Miltenyi Biotec; 130‐094‐183).

### Flow Cytometry Analysis of CD8^+^CXCR6^+^T Cells

CD8^+^CXCR6^+^T and CD8^+^CXCR6^‐^T were enriched with flow cytometry. Prostates were minced and digested at 37 °C for 45 min in media containing DMEM supplemented with 1% FBS, 1% glutamine, 1% non‐essential amino acids, 1% sodium pyruvate, 1% penicillin/streptomycin, 10 mM HEPES (Invitrogen Life Technologies), 0.2% collagenase II (Gibco, 17101015), 2.5 U mL^−1^ Dispase II (Sigma‐Aldrich, D4693). Intracellular staining for GZMB and PRF1 were ducted using BD Cytofix/Cytoperm and BD Perm/Wash buffers (BD Biosciences, 554714) according to the manufacture's recommendations. To detect intracellular GZMB and PRF1, the enriched cells isolated from prostate cancer tissues were incubated with 50 ng mL^−1^ PMA (Sigma‐Aldrich), 500 ng mL^−1^ ionomycin (Sigma‐Aldrich), and 10 mg mL^−1^ brefeldin A (Sigma‐Aldrich) for 4 h at 37 °C and stained as described earlier. Samples were analyzed on a CytoFLEX S platform (Beckman Coulter) and FlowJo V10.4 software. The antibodies used were as follows: Alexa 488 conjugated anti‐human CD8 (Cat. No. 557704; BD Biosciences), PE anti‐human CD186 (CXCR6) (Cat. No. 356004; Biolegend), and BV421 anti‐human GZMB (BD Biosciences; 563389), BV421 anti‐human PRF1(BD Biosciences; 563393), and LIVE/DEAD Fixable Violet Dead Cell Stain Sampler Kit (ThermoFisher, L34964).

### Flow Cytometry Analysis of CD4^+^Foxp3^+^Treg

After co‐culturing 3 days with CAFs, cells were collected and washed twice with cold PBS. Then cells were then pre‐incubated with LIVE/DEAD Fixable Violet Dead Cell Stain Sampler Kit (ThermoFisher, L34964) for 30 min at 4 °C. After surface antigens (CD3e, CD4) staining, cellular fixation, and permeabilization, Foxp3 staining was performed according to the manufacturer's protocol (BD, transcription factor Buffer set, 562574). Flowjo V10.4 software was used for data analysis. The antibodies used were as follows: PE anti‐mouse CD4 (Cat. No. 100408; Biolegend), APC‐Cy7 anti‐Mouse CD3e (Cat. No. 557596; BD Biosciences), and Alexa Fluor 488 Rat anti‐Mouse Foxp3 (BD Biosciences, 560403). The flow cytometry analysis diagram and gating strategy were illustrated in Figure 7J and Figure S11F in Supporting Information, respectively.

### Hematoxylin–Eosin (H&E), Immunofluorescence and Immunohistochemistry (IHC) Staining

Prostates were fixed in 4% formaldehyde overnight at 4 °C and embedded either in paraffin or OCT for the paraffin section or frozen section, respectively. Sections were stained with hematoxylin and eosin and antibodies against human AR (Cat. No. ab133273; Abcam), NKX3‐1 (Cat. No. ab196020; Abcam), P63 (4A4) mouse monoclonal primary antibody (Roche), KLK3 (Abclonal, A2052), Ki‐67 (Cat. No. 12202; Cell Signaling Technology), KLK12 (bs‐5865R, Bioss), CD86 antibody (Cat. No. ab269587; Abcam), CD68 (Cat. No. ab955; Abcam), beta Catenin (Cat. No. ab22656; Abcam), and Synaptophysin antibody [YE269] (Cat. No. ab32127, Abcam). IHC staining was performed using standard protocols.

The protocol for immunofluorescence staining resembled IHC staining but without an antigen retriever. The antibodies used for immunofluorescence staining were as follows: CD8 (Invitrogen, MA1‐81692), CXCR6 (Cat. No. ab8023; Abcam), GZMB (Invitrogen, MA1‐80734), SOX9 (Abclonal, A19710), KLF5 (Abclonal, A2989), beta‐Catenin (Cat. No. ab22656; Abcam), Cytokeratin 13 antibody [AE8] (Cat. No. ab16112; Abcam), FAP (Cat. No. ab207178; Abcam), FOXP3 (Cat. No. ab20034; Abcam), Donkey anti‐mouse IgG Alexa Fluor 488 (Invitrogen, A‐21202), Donkey anti‐rabbit Alexa Fluor 555 (Invitrogen, A‐31572), Donkey anti‐Rat IgG Alexa Fluor 488 (Invitrogen, A‐21208), Donkey Anti‐mouse Alexa Fluor 555 (Invitrogen, A‐31570), Goat Anti‐Rabbit IgG Alexa Fluor 488 (Cat. No. ab150077; Abcam) and Donkey Anti‐mouse Alexa Fluor 647 (Cat. No. ab150107; Abcam).

### Western Blot Analysis

For Western blotting, proteins were extracted from cell lysates using RIPA buffer (Thermo Scientific, 89900) plus with Halt Phosphatase Inhibitor Cocktail (Thermo Scientific, 78420) and measured with a Pierce BCA Protein Assay kit (Thermo Scientific, 23225). The antibodies used for Western blot assay were as follows: SOX9 (Abclonal, A19710), KLF5 (Abclonal, A2989), ELF3 (Abclonal, A5236), MYC (Abclonal, A19032), CTNNB1(Abclonal, A19657), WNT7A (Abclonal, A5425), TMPRSS2 (Abclonal, A9126), KRT13 (Abclonal, A0411). Goat anti‐Rabbit IgG (H+L) Secondary Antibody, HRP (Invitrogen, 65–6120). Dilutions of all primary antibodies were 1:1000. Dilutions of the secondary antibody were 1:5000.

### Chromatin Immunoprecipitation (ChIP) and ChIP‐ Sequencing (ChIP‐Seq) Assay

The ChIP assay was performed according to the manufacturer's protocol (Abclonal, RK20258). The ChIP kit used were as follows: Sonication ChIP Kit (Abclonal, RK20258), Protein A/G Beads (Abclonal, RM02915) protease inhibitor (Abclonal, RM02916). Briefly, cells were washed with 1X PBS and fixed with formaldehyde at a final concentration of 1% for 10 min at room temperature. Cross–linking was quenched by the addition of a final concentration of 0.125 m glycine for 5 min at room temperature. Cells were then rinsed and lysed in 1 mL cell lysis buffer for 10 min on ice with protease and phosphatase inhibitors. Then, the samples were pelleted and resuspended in 1 mL nuclear lysis buffer, and sonicated to obtain chromatin fractions from 200 to 600bp using a water bath sonicator (Diagenode, Liege, Belgium). After preclearing with a 50% slurry of protein A‐G beads preincubated with salmon sperm DNA and bovine serum albumin for 4–6 h at 4 °C, the chromatins were incubated with 5ug ChIP‐grade antibody or 1 ug normal rabbit IgG overnight. The antibody‐bound chromatin was then pulled down for 3 h with protein A‐G beads, washed extensively (low salt wash buffer, high salt wash buffer, LiCl wash buffer, and TE wash buffer), and eluted two times with Elution buffer. After the addition of 8 µl of 5 m NaCl, the decross–linking 4 h (or overnight) incubation at 65 °C. The immunoprecipitated DNAs as well as whole cell extract DNAs (Input) were preliminarily purified by treatment with RNase A and then proteinase K followed by further purification. The purified DNA was used for CHIP‐PCR analyses using the relevant primers for CTNNB1. ChIP Antibodies: SOX9 (Abclonal, A19710), KLF5 (Abclonal, A2989), ELF3 (Abclonal, A5236). The CTNNB1 primer sequences were as follows: CTNNB1 promoter forward: 5'‐ CTCAGACGGCAGCAGACT‐3', CTNNB1 promoter reverse: 5'‐ TTAAAATGGCGCCGCACAA‐3'.

### ChIP‐Seq Analysis

The ChIP‐seq libraries were sequenced on the Illumina sequencing platform by Novogene Co., Ltd (Beijing, China). The main steps are listed below:

### Sample Collection and Preparation

1. DNA quantification and qualification: ChIP DNA degradation and contamination were monitored on agarose gels. DNA purity was checked using the NanoPhotometer spectrophotometer (IMPLEN, CA, USA). DNA concentration was measured using a Qubit DNA Assay Kit in a Qubit 3.0 Fluorometer (Life Technologies, CA, USA).
2. Library preparation and quantification: The purified DNA was used for ChIP‐seq library preparation. The library was constructed by Novogene Corporation (Beijing, China). Subsequently, pair‐end sequencing of the sample was performed on Illumina platform (Illumina, CA, USA). Library quality was assessed on the Agilent Bioanalyzer 2100 system.

### Data Analysis—Quality Control

Raw data (raw reads) of fastq format were first processed using fastp (version 0.19.11) software.^[^ ^50^ ^]^ In this step, clean data (clean reads) were obtained by removing reads containing adapter, reads containing ploy‐N and low‐quality reads from raw data. At the same time, Q20, Q30, and GC content of the clean data were calculated. All the downstream analyses were based on clean data with high quality.

### Data Analysis—Reads Mapping to the Reference Genome

Reference genome and gene model annotation files were downloaded from genome website directly. Index of the reference genome was built using BWA (v 0.7.12) and clean reads were aligned to the reference genome using BWA mem (v 0.7.12).

### Data Analysis—Fragment Size Estimation

For a specific ChIP‐seq binding site, individual reads mapping to the plus or minus strand and present significant enrich. For single‐end sequencing, the fragment size was estimated with MACS2^[^ ^49^ ^]^ predicted method with default parameters. MACS2 scans all the genome with a specific window size and calculates the reads enrichment level. A particular number of windows were used as samples to build an enrichment model and to predict fragment size. The following peak calling analysis was based on this predicted fragment size.

### Data Analysis—Peak Detection

After mapping reads to the reference genome, the MACS2 (version 2.1.0) peak‐calling software was used to identify regions of IP enrichment over background. A *q*‐value threshold of 0.05 was used for all data sets. After peak calling, the distribution of chromosome distribution, peak width, fold enrichment, significant level, and peak summit number per peak were all displayed.

### Data Analysis—Motif Analysis

Homer^[^ ^51^ ^]^ was used to detect the denovo sequence motif and the matched known motifs.

### Statistics

All data were analyzed using GraphPad Prism 9.0. The significance of the differences between different groups was evaluated using unpaired two‐tailed Student's t‐tests or Mann–Whitney *U‐*test. All values were expressed as the mean ± standard deviation, and significance was set at *p <* 0.05 (*p < 0.05, **p < 0.01; ***p < 0.001; ****p < 0.0001).

## Conflict of Interest

The authors declare no conflict of interest.

## Author Contributions

X.B., W.W., and M.A. contributed equally to this work. J.W., Y.Z., M.Z., and D.Y. conceived the study. X.B., W.W., M.A, and M.Z performed the bioinformatics analysis, ChIP and Western blot experiments, and contributed to the data analysis. G.S., X.Z., L.D., W.M., and W.W. performed the cell culture, FACS and IHC/IF experiments. M.X., X. W., H. S., C.T., and C.Z. provided suggestions and helped with sample collection. X.H download the processed expression data (https://singlecell.broadinstitute.org/single_cell/study/SCP864). J.W., W.W., M.Z, and X.B. wrote and revised the manuscript. All authors read and approved the final manuscript.

## Supporting information

Supporting Information

## Data Availability

The ScRNA‐Seq data, spatial transcriptomics data, ChIP‐seq data, and bulk ATAC‐Seq data reported in this study were deposited at the Genome sequence Archive Family^[^ ^52^ ^]^ (GSA for Human: the accession number: HRA004818, BioProject: the accession number: PRJCA017621).
