# Supplementary material for: Integration Analysis of Single‐Cell Multi‐Omics Reveals Prostate Cancer Heterogeneity
Source: Adv Sci (Weinh). 2024 Mar 14;11(18):2305724. doi: 10.1002/advs.202305724 (PMC11095148; doi:10.1002/advs.202305724)
Supplement: Supplementary file 1 — Supporting Information [file ADVS-11-2305724-s001.pdf]

ADVANCED SCIENCE
Open Access

Supporting Information

for *Adv. Sci.*, DOI 10.1002/advs.202305724

Integration Analysis of Single-Cell Multi-Omics Reveals Prostate Cancer Heterogeneity

Xiaojie Bian, Wenfeng Wang, Mierxiati Abudurexiti, Xingming Zhang, Weiwei Ma, Guohai Shi, Leilei Du, Midie Xu, Xin Wang, Cong Tan, Hui Sun, Xiadi He, Chenyue Zhang, Yao Zhu, Min Zhang*, Dingwei Ye* and Jianhua Wang**

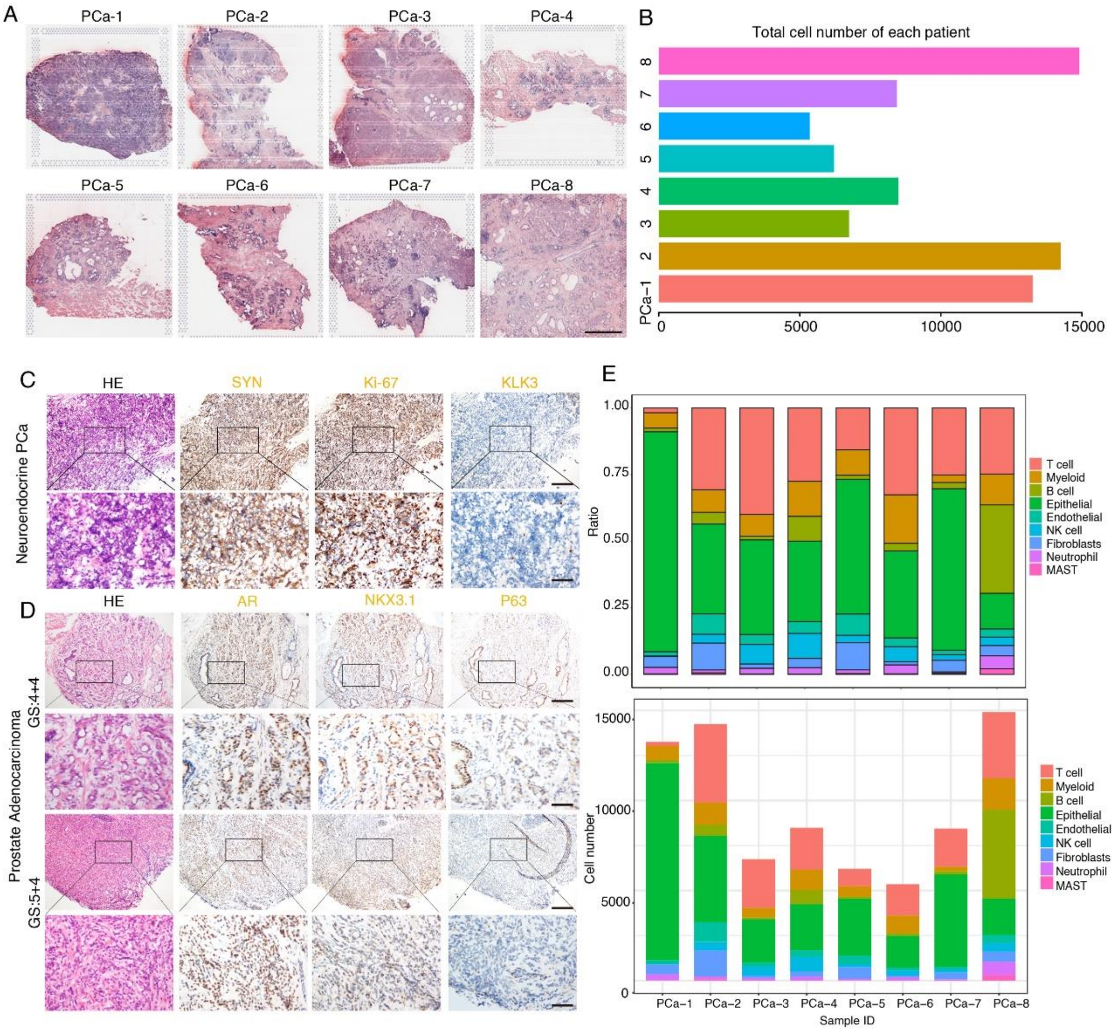

Figure S1. Overview the histopathological morphology and cell composition of each patient.

A. H&E staining to illustrate the of histopathological morphology changes in each patient. Scale bar: 200μm.

B. Total cell number in each patient after quality control and filtering.

C-D. IHC staining results showed the marker gene expression in neuroendocrine prostate cancer as well as the prostate adenocarcinoma. The NEPC patient highly expressed SYN and Ki-67, while loss the expression of PSA (KLK3). In contrast, the prostate adenocarcinoma patients expressed luminal cell markers AR and NKX3-1. P63 (TP63) indicted the residue basal cells in prostate adenocarcinoma patients. Scale bar: 200μm (upper panel), 50μm (lower panel).

E. Cell composition illustrated in ratio (upper panel) and absolute cell number (lower panel) in each patient.

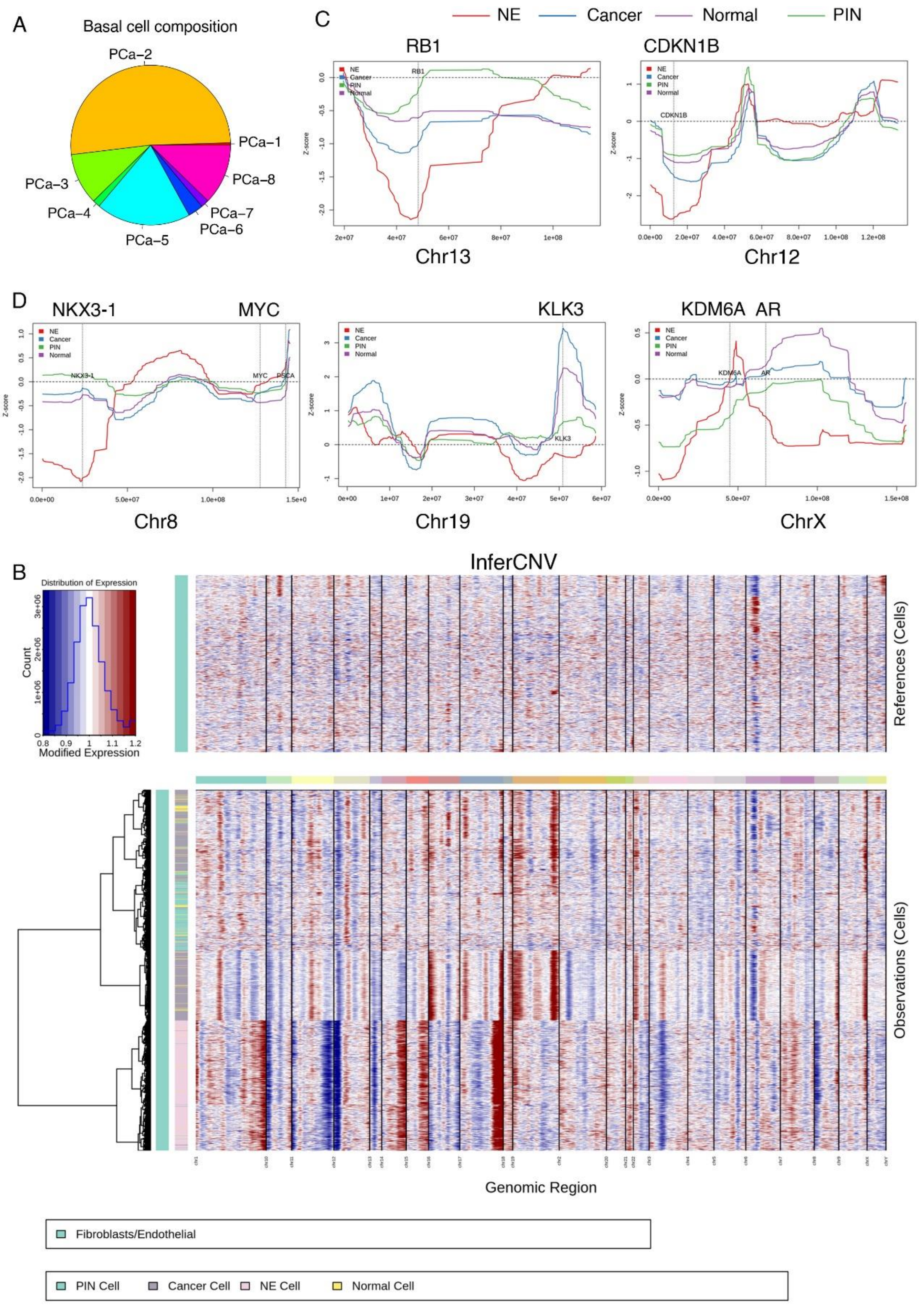

Figure S2. Investigate the copy number variation in epithelial cells using InferCNV as well as the common genetic alterations using Z-score.

A. Pie plot to show the relative basal cell numbers in each patient.

B. InferCNV result (heatmap) to illustrates the relative expression intensities across each

chromosome. NE cells show the most CNV difference from other cell types. In contrast, the normal epithelial cells do not show noticeable difference from the reference cells. NE: NEPC, Cancer: the other epithelial cells which were annotated as tumor cells; Normal: Epithelial cells annotated as “Normal epithelia” cells; PIN: the pre-cancerous lesion/ Epithelial cells annotated as normal-like epithelial cells.

C and D. Investigate the common genetic alterations in prostate cancer using Z-score.

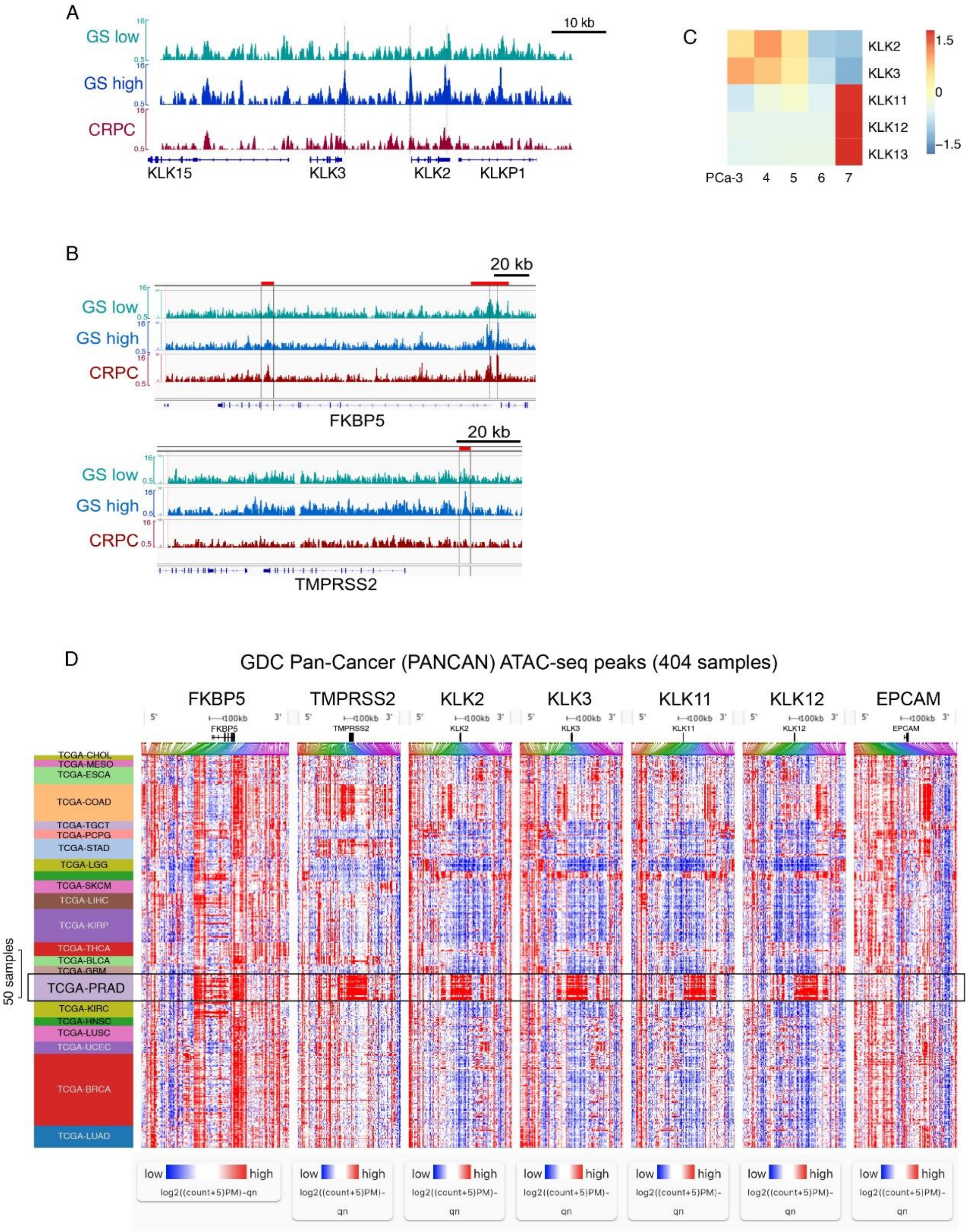

Figure S3. Dissect the chromatin accessibility changes as PCa progression.

A and B. ATAC-seq peak enrichment in AR target genes, KLK2, KLK3, TMPRSS2 and FKBP5 gene loci in our data.

C. bulk RNA-Seq results suggested KLK11 and KLK12 expressed robustly in the CRPC patient compared with the other patients.

D. Pan-cancer ATAC-seq dataset illustrate the ATAC peak enrichment of AR target genes, including FKBP5, TMPRSS2, KLK2, KLK3, KLK11, and KLK12 in prostate samples.

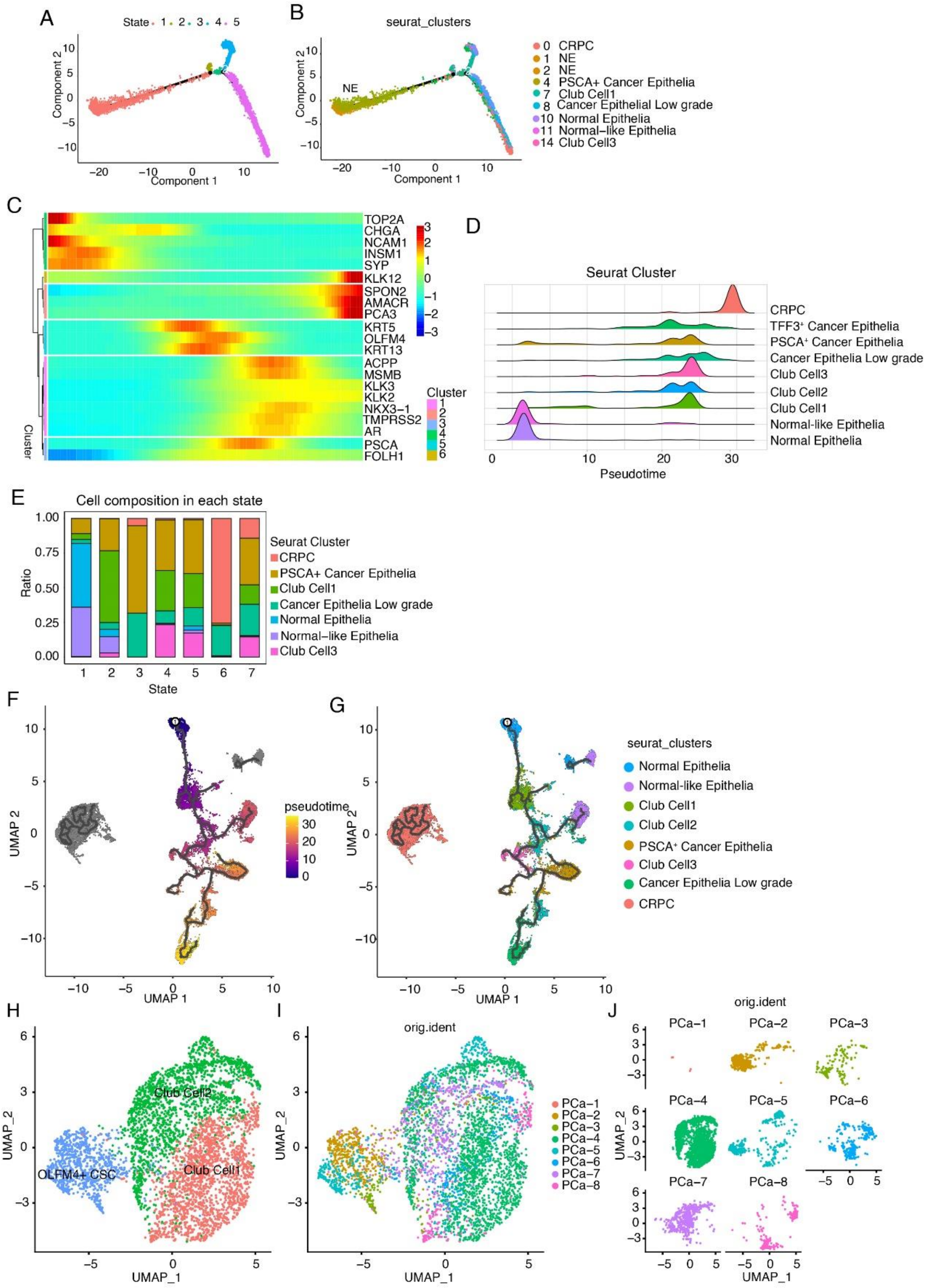

Figure S4. Dissect the epithelia subset distribution *in situ* in each patient.

A-B. Pseudotime cell trajectories analysis using Monocle 2 to study the epithelial cellular dynamic changes in each state. Visualization in state (A) or Seurat cluster (B). NE cells showed a distinct transcriptome profile that disconnected from other luminal cells.

C. Pseudotime heatmap to visualize the modules of genes of total epithelia that co-vary across the pseudotime. Those highly expressed genes in the start state are NE marker genes, such as CHGA, NCAM1 and INSM1.

D. Ridge plot to show the epithelial cellular dynamic changes without NE cells. The start sate is normal epithelia.

E. Cellular composition in each state. State 1 is mainly composed of “Normal” or “Normal-like” epithelia, while the state 6 is mainly composed of “CPRC” epithelia subset.

F and G. The same cell clusters as for the Monocle 2 analysis were selected and reanalyzed using Monocle 3. The reanalysis trajectories result using Monocle 3 were very similar to Monocle 2. Visualization in pseudotime (F) or Seurat cluster (G).

H-J. Illustrate the club cell sample origin side-by-side in cell type (H), group by original identity/sample origin (I), and split by sample identification (J).

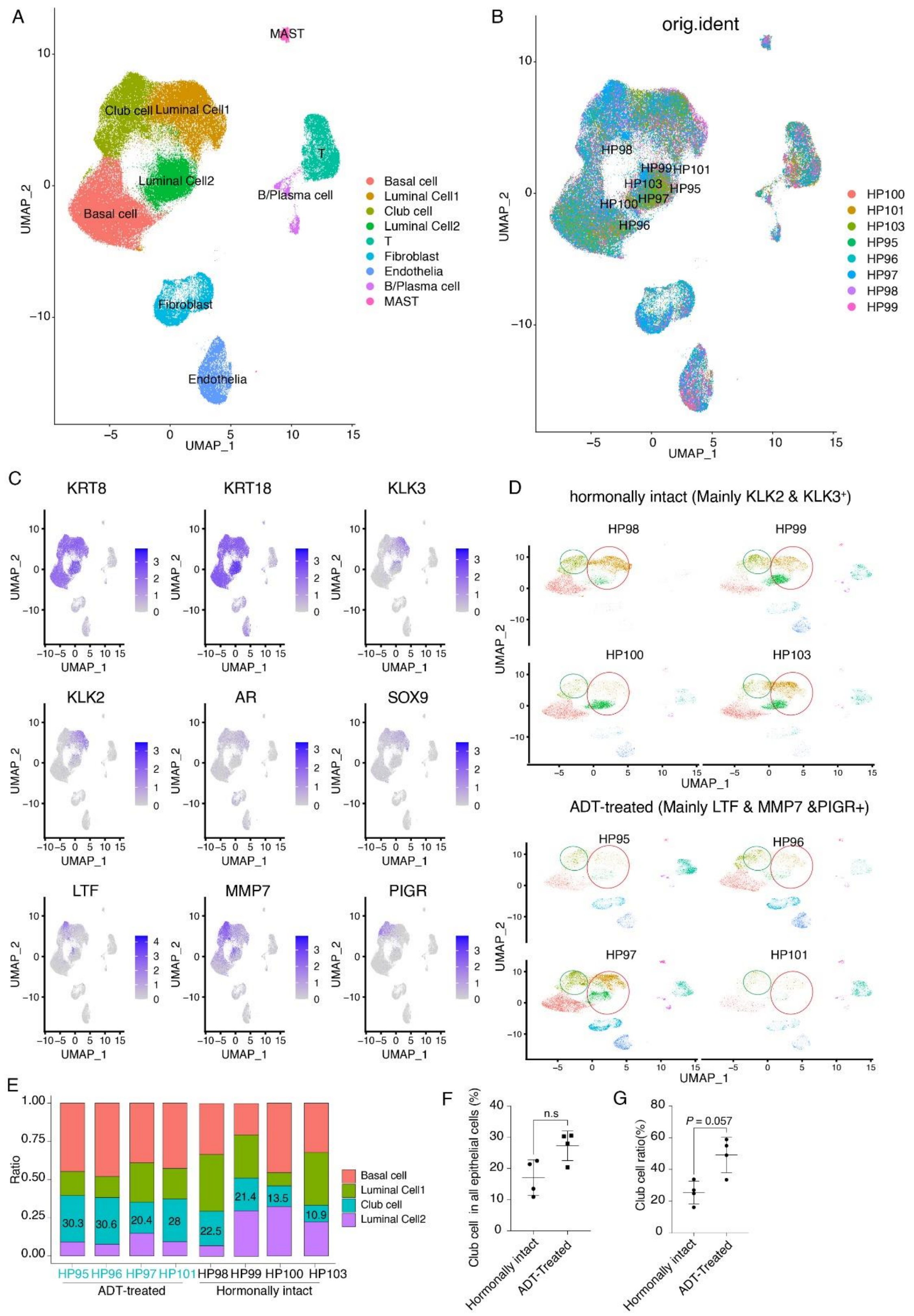

Figure S5. ADT-treated induces dramatic loss of luminal cells and club cells enriched after treatment.

A and B. UMAP plot to illustrate the main cell types (A) or group by their original identities (B). Harmony is used to remove batch effects.

C. Feature plot to illustrate the luminal cell marker or club cell marker gene expression. KLK2, KLK3 and AR are well-known luminal cell markers; LTF, MMP7 and PIGR are canonical club cell markers.

D. HP98, HP99, HP100 and HP103 are hormonally intact samples, HP95, HP96, HP97 and HP101 are ADT-treated samples. Red circles indicated the luminal cell subsets, green circles indicated the club cell subsets.

E. The relative cellular composition in each patient. The numbers labeled in the stack graph indicate the relative ratio of club cells.

F. Club cell ratio in all epithelial cells. Compared to the hormonally intact group, there is an increasing trend in the ADT-treated group. Mann-Whitney *U* Test, *P*-value = 0.11.

G. Compared to the hormonally intact group, the club cell ratio was significantly increased in the ADT-treated group (after elimination of basal cells). Mann-Whitney *U* Test, *P*-value = 0.057.

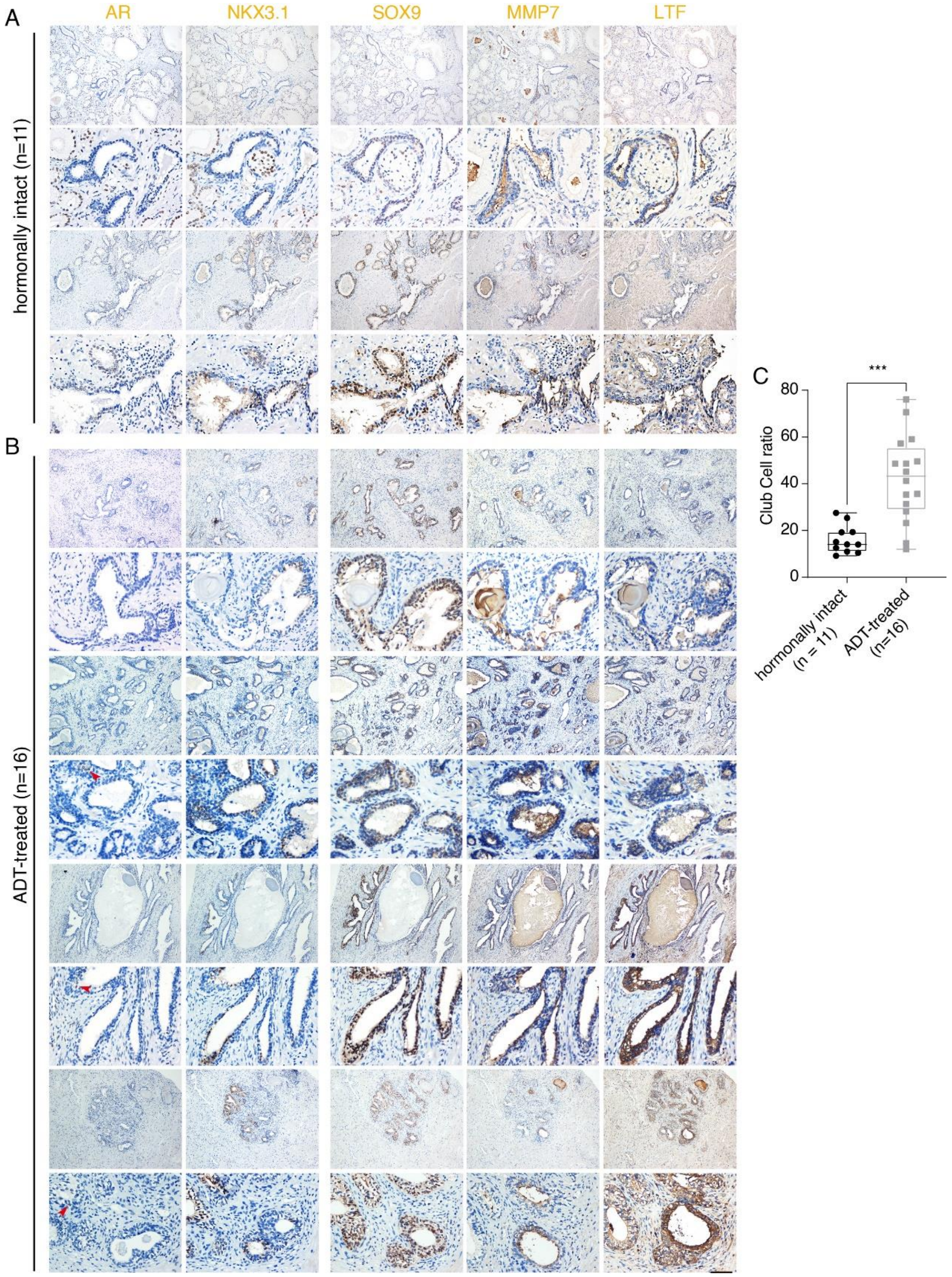

Figure S6. IHC staining demonstrate that the majority of residual morphologically "normal" cells are actually club cells.

A and B. Representative IHC images from 11 hormonally intact (A) and 16 ADT-treated samples (B). AR and NKX3-1: luminal cell marker, MMP7 and LTF: club cell marker. Red arrow indicated the persistent AR-positive luminal cells after ADT. Scale bar: 50μm.

C. Statistics results of club cell ratio in epithelial cells. Mann-Whitney *U* Test, ***$P < 0.01$.

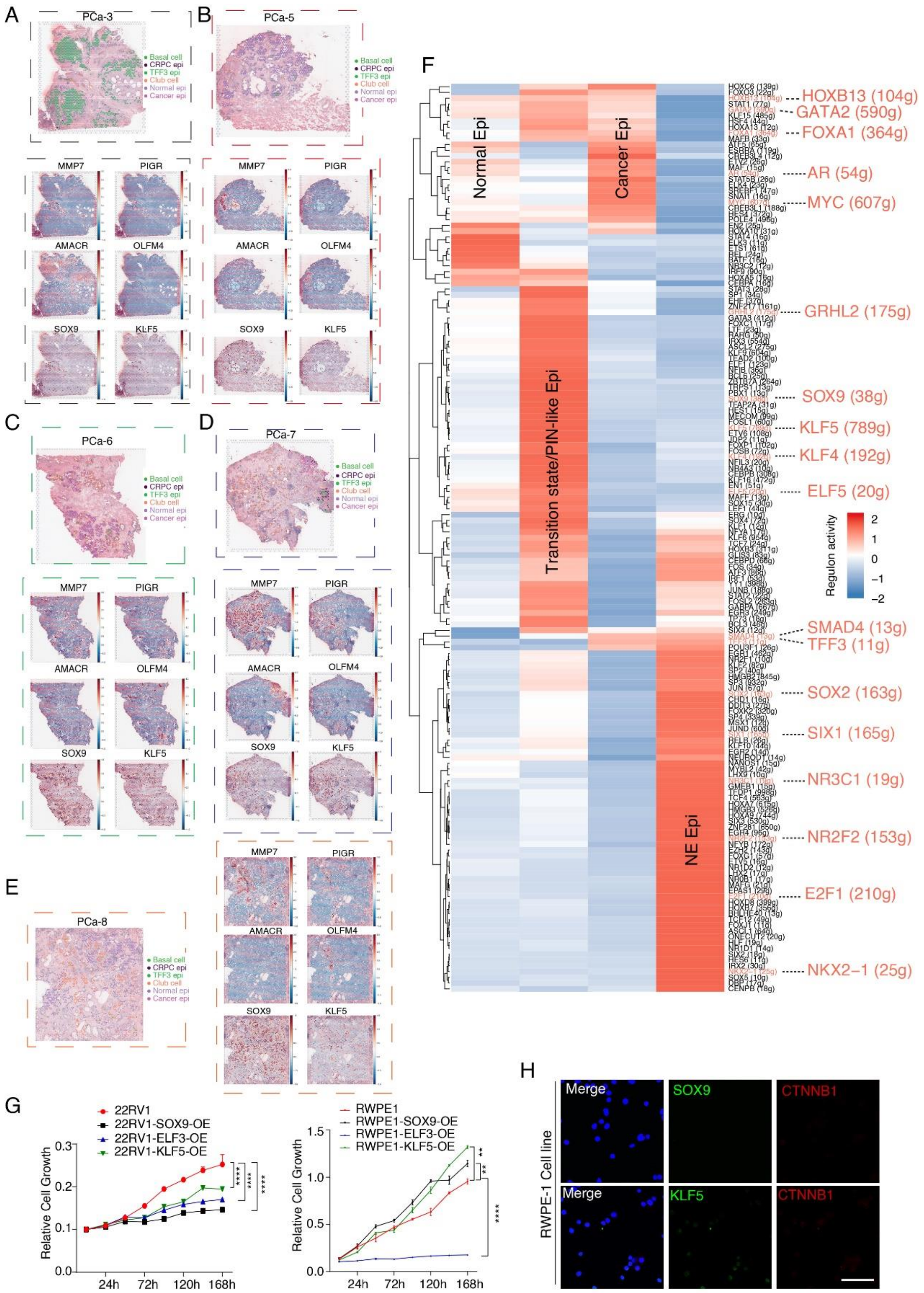

Figure S7. Spatial distribution of epithelial subsets and investigate the transcriptional factors that drive the differentiation of epithelial cells using SCENIC analysis.

A-E. Clustering analysis of spatial transcriptomic data revealed the epithelia subset distribution *in situ* in PCa-3, PCa-5 to PCa-8.

F. SCENIC analysis results suggested both normal and cancer epithelia share the canonical luminal transcription factors (TFs) such as *HOXB13*, *GATA2*, and *AR*, while SOX2, *SIX1* and *NKX2-1* regulon upregulated significantly in NE cells.

G. CCK-8 results found upregulation of SOX9 or KLF5 inhibits the growth of 22Rv1 cell line but promotes the growth of RWPE-1 cell line.

H. Wnt signaling is not activated under normal conditions in RWPE-1 cell line. Scale bar: 50μ m.

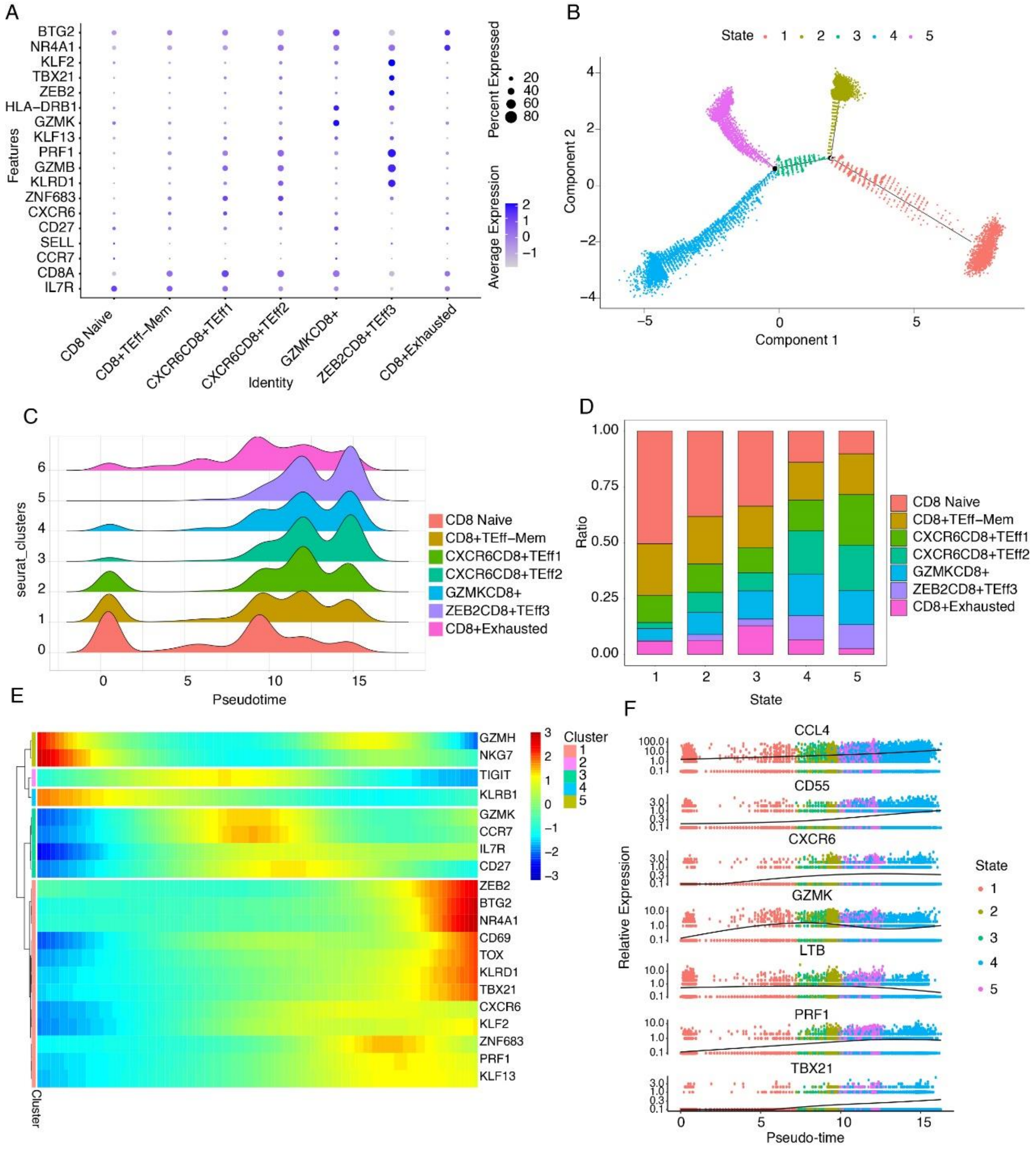

Figure S8. Delineate the tumor-infiltrating CD8^{+}T cell subset in the prostate cancer microenvironment

A. Dot plot shows the marker genes expression pattern CD8^{+}T subset. Here we subdivided CD8^{+}T cells into 7 subpopulations. Naïve CD8^{+}T marked with *CCR7* and *SELL* expression; effector CD8^{+}T cells marked with *GZMB*, *PRF1* and *LAMP1*(CD107a) expression. The exhausted CD8^{+}T subset lost the expression of cytotoxic granules but highly expressed the exhaustion marker *NR4A1* and *BTG2*.

B-C. Pseudotime cell trajectories analysis using Monocle 2 to study the CD8^{+}T cellular dynamic processes visualization in state (B) or pseudotime (C). Compared with effector CD8^{+}T, Naïve CD8^{+}T showed similar transcriptional characteristics with exhausted CD8^{+}T.

D. The ratio of $CD8^+$T cell composition in each state.

E. Pseudotime cell trajectories analysis using Monocle 2 to study the $CD8^+$T cellular dynamic processes visualization in heatmap.

F. Naïve to effector switch concurrent with a continuous activation of *CXCR6*, *PRF1* and *TBX21*.

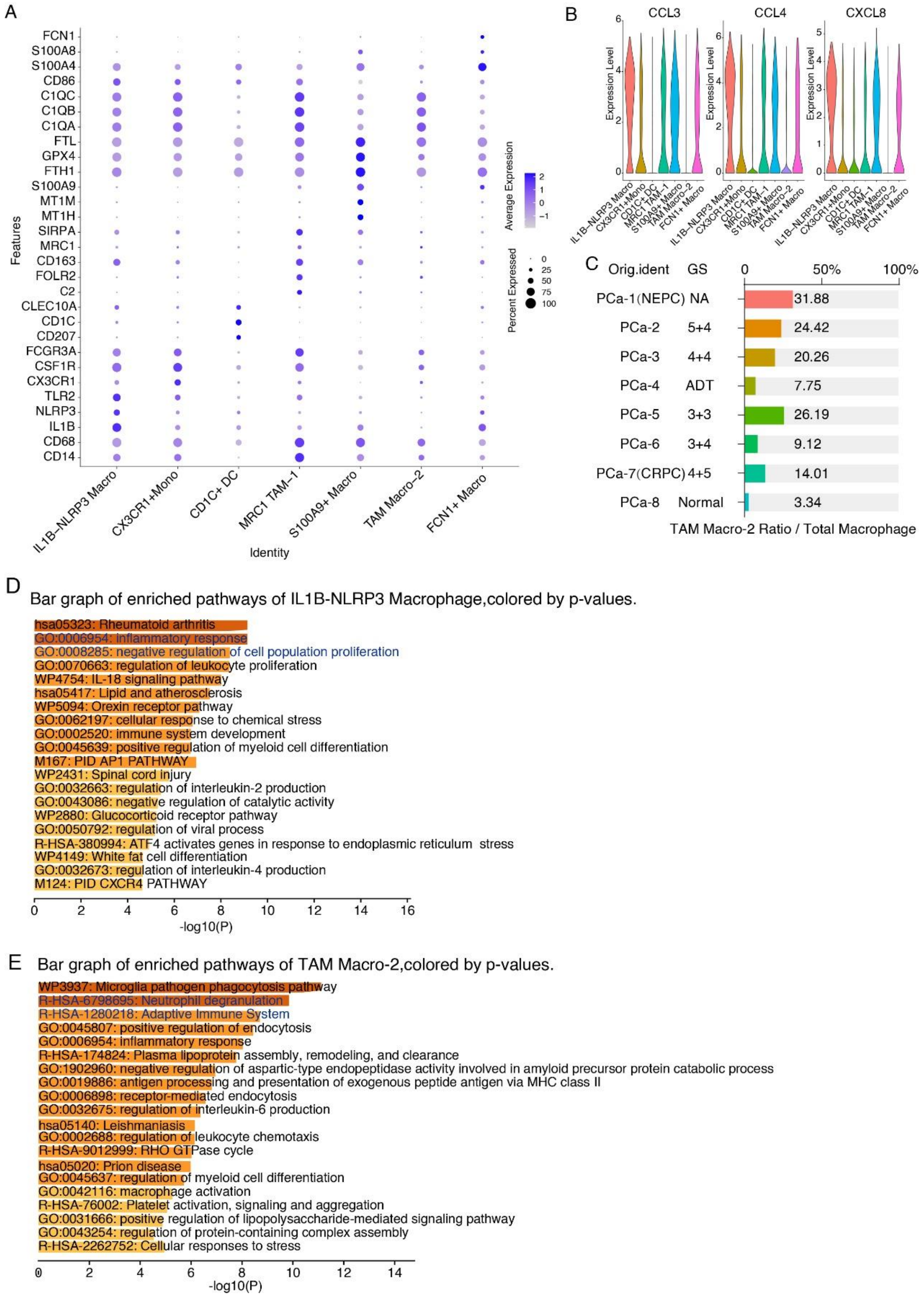

Figure S9. Delineate the tumor-infiltrating myeloid cells subset in the prostate cancer microenvironment

A. Dot plot shows the marker genes expression pattern myeloid cells subset. Myeloid cells, including monocyte, macrophage, and dendritic cells, were categorized into 7 cell types by

scRNA-seq.

B. The TAM Macro-2 subset does not secrete T-cell chemotactic factors *CCL3*, *CC*L4 or *CXCL8.*

C. The TAM Macro-2 infiltrated abundance in cancer patients.

D-E. Compare the signaling pathway difference between the IL1B-NLRP3 Macro subset (D) with TAM Macro-2 subset (E). All gene ontology (GO) analyses were performed with the online tool–Metascape (http://metascape.org)[1].

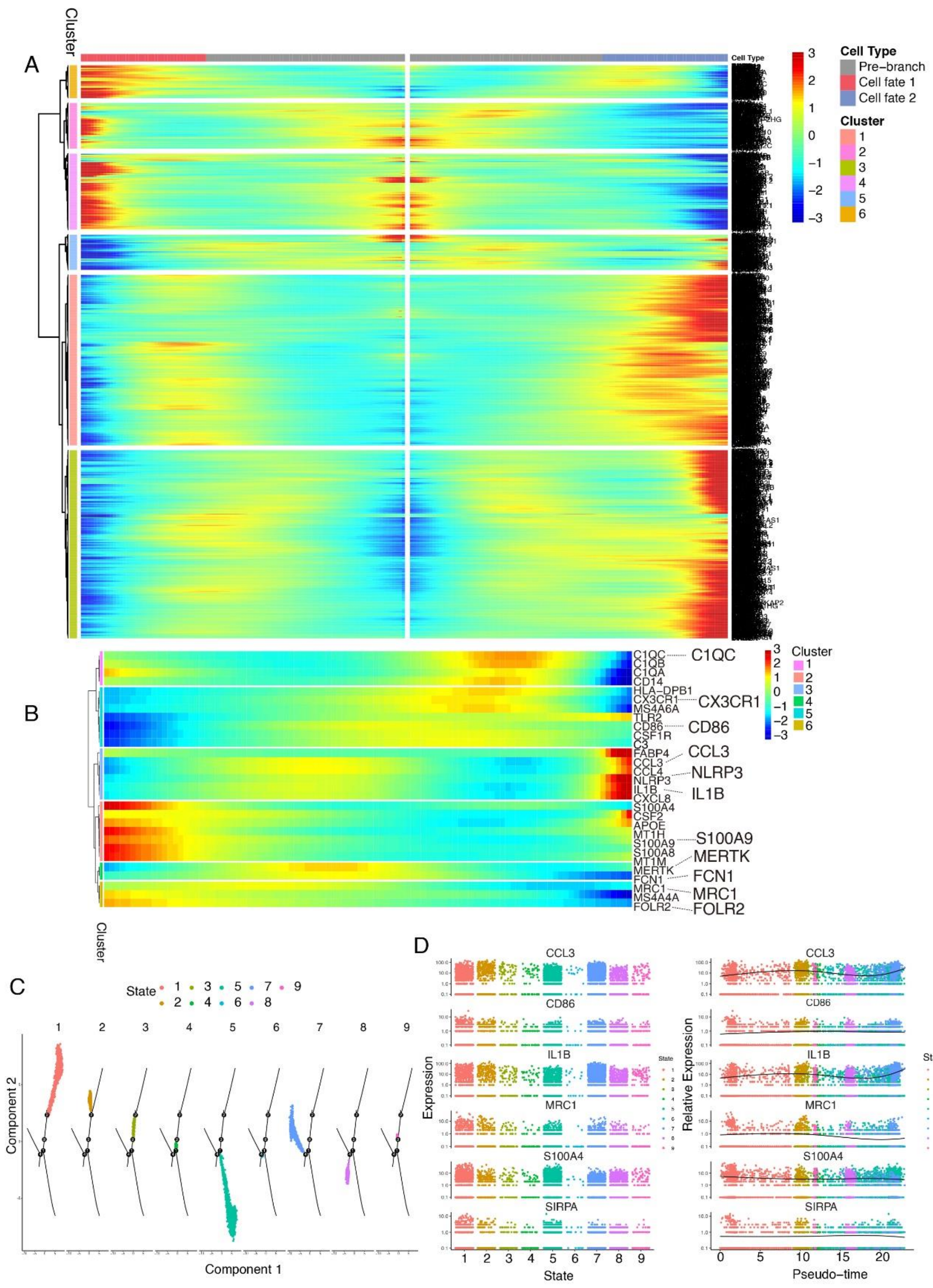

Figure S10. BEAM analysis in Monocle2 and pseudotime heatmap to visualize the modules of genes that co-vary across the state transition.

A. BEAM branch analysis using Monocle2 to find genes associated with branching in macrophage fate decisions. We use the plot_genes_branched_heatmap function and the parameter qval < 1e-4.

B. Pseudotime heatmap to visualize the modules of genes that co-vary across the state transition.

C. Pseudotime analysis result demonstrated each state in facet_wrap.

D. Immune trafficking chemokines (CCL3 and IL1B) and immunosuppressive markers (MRC1 and SIRPA) changed dramatically across the pseudotime.

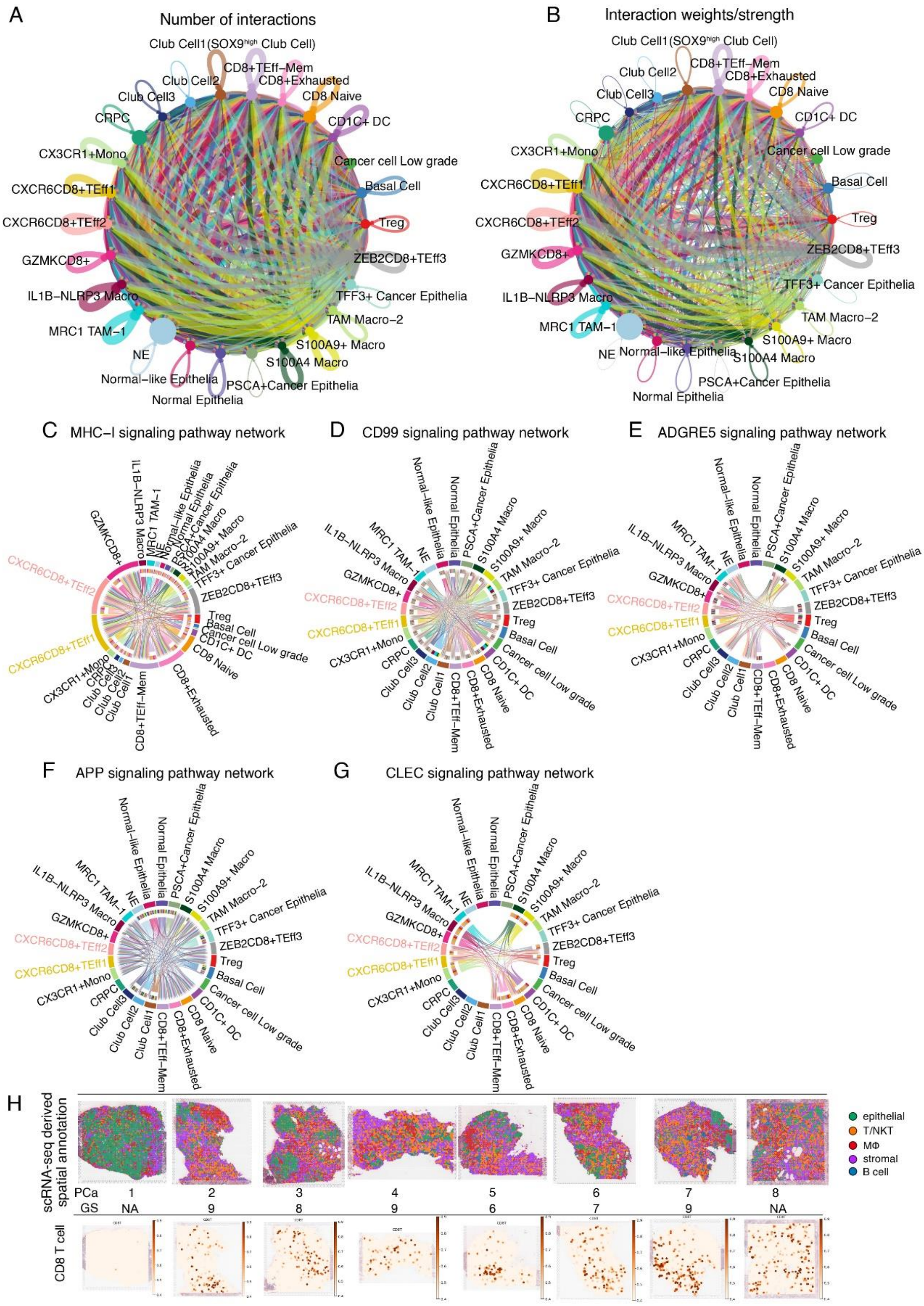

A
Number of interactions
B
Interaction weights/strength
C
MHC−I signaling pathway network
D
CD99 signaling pathway network
E
ADGRE5 signaling pathway network
F
APP signaling pathway network
G
CLEC signaling pathway network
Club Cell1(SOX9high Club Cell)
CD8+TEff−Mem
CD8+Exhausted
CD8 Naive
CD1C+ DC
Cancer cell Low grade
Basal Cell
Treg
ZEB2CD8+TEff3
TFF3+ Cancer Epithelia
TAM Macro−2
S100A9+ Macro
S100A4 Macro
PSCA+Cancer Epithelia
Normal Epithelia
Normal−like Epithelia
NE
MRC1 TAM−1
IL1B−NLRP3 Macro
GZMKCD8+
CXCR6CD8+TEff2
CXCR6CD8+TEff1
CX3CR1+Mono
CRPC
Club Cell3
Club Cell2
H
scRNA-seq derived spatial annotation
PCa 1 2 3 4 5 6 7 8
GS NA 9 8 9 6 7 9 NA
CD8 T cell
epithelial
T/NKT
MΦ
stromal
B cell

Figure S11. Cell-cell communications are shown among the Myeloid-Epithelial-T cell subsets.

A-B. Circle plot of the overall cell–cell communication within the Myeloid-Epithelial-T cell types calculated by CellChat. A and B indicated the number of interactions (A) and interaction weights/strength (B), respectively.

C-G. $CD8^+CXCR6^+$ T communicate with myeloid cells and epithelial cells via MHC-I singling pathway (C), CD99 signaling pathway (D), ADGRE5 signaling pathway (E), APP signaling pathway (F) and CLEC signaling pathway (G).

H. Dissected the $CD8^+$T cell distribution *in situ* in each patient. Upper panel: Spatial annotation of the main cell types of each patient. Lower panel: Dissected the $CD8^+$T cell distribution *in situ* in each patient.

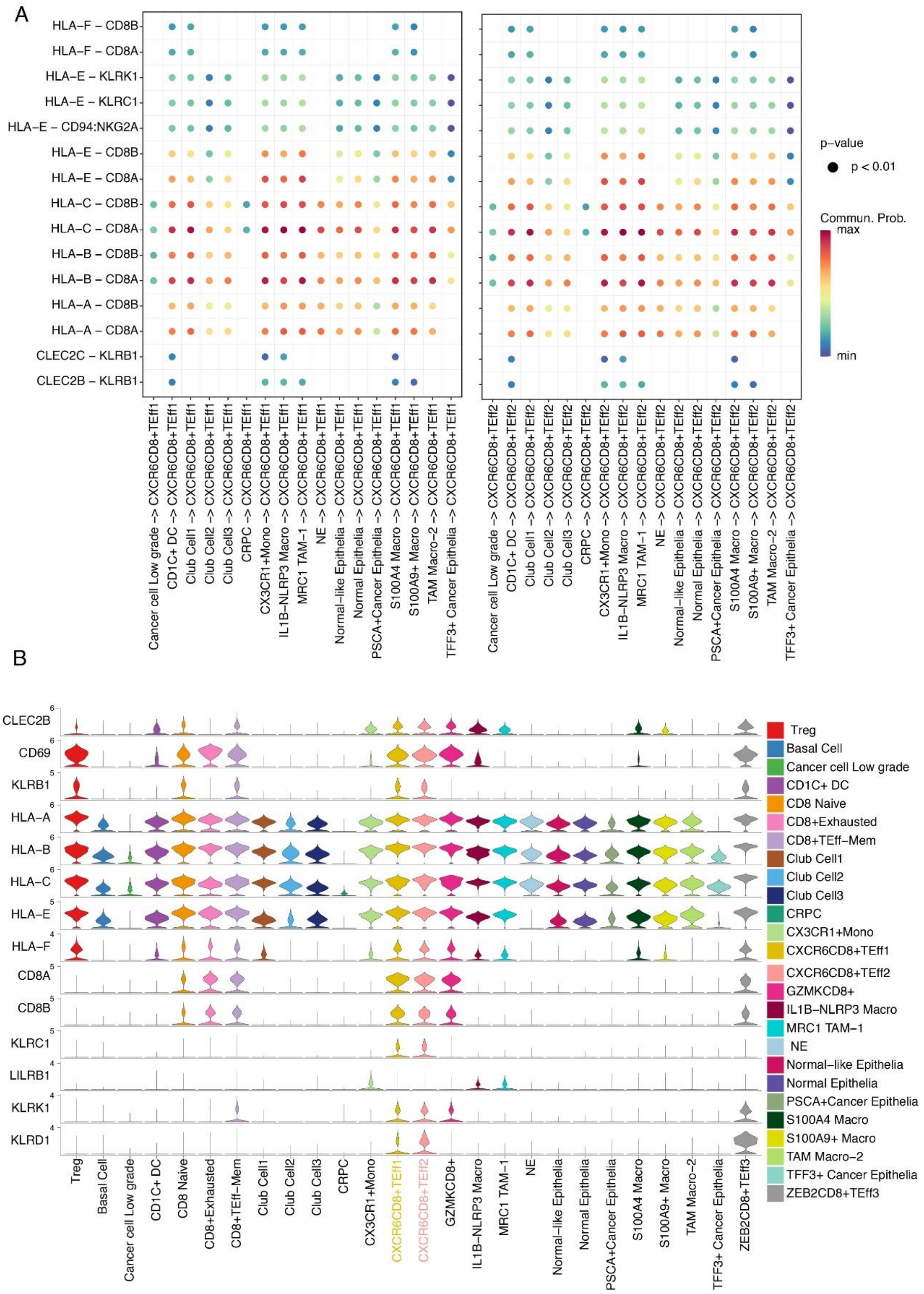

Figure S12. CLEC2B/2C-KLRB1 signaling pathways may be the prominent pathways which shape the CD8^{+}CXCR6^{+}T in anti-cancer responses.

A. Visualize the cell-cell communication/ligand-receptor pairs among CD8^{+}CXCR6^{+}T cells and other cell types in bubble plot.

B. Visualize the gene expression level in CLEC signaling pathways using the CellChat.

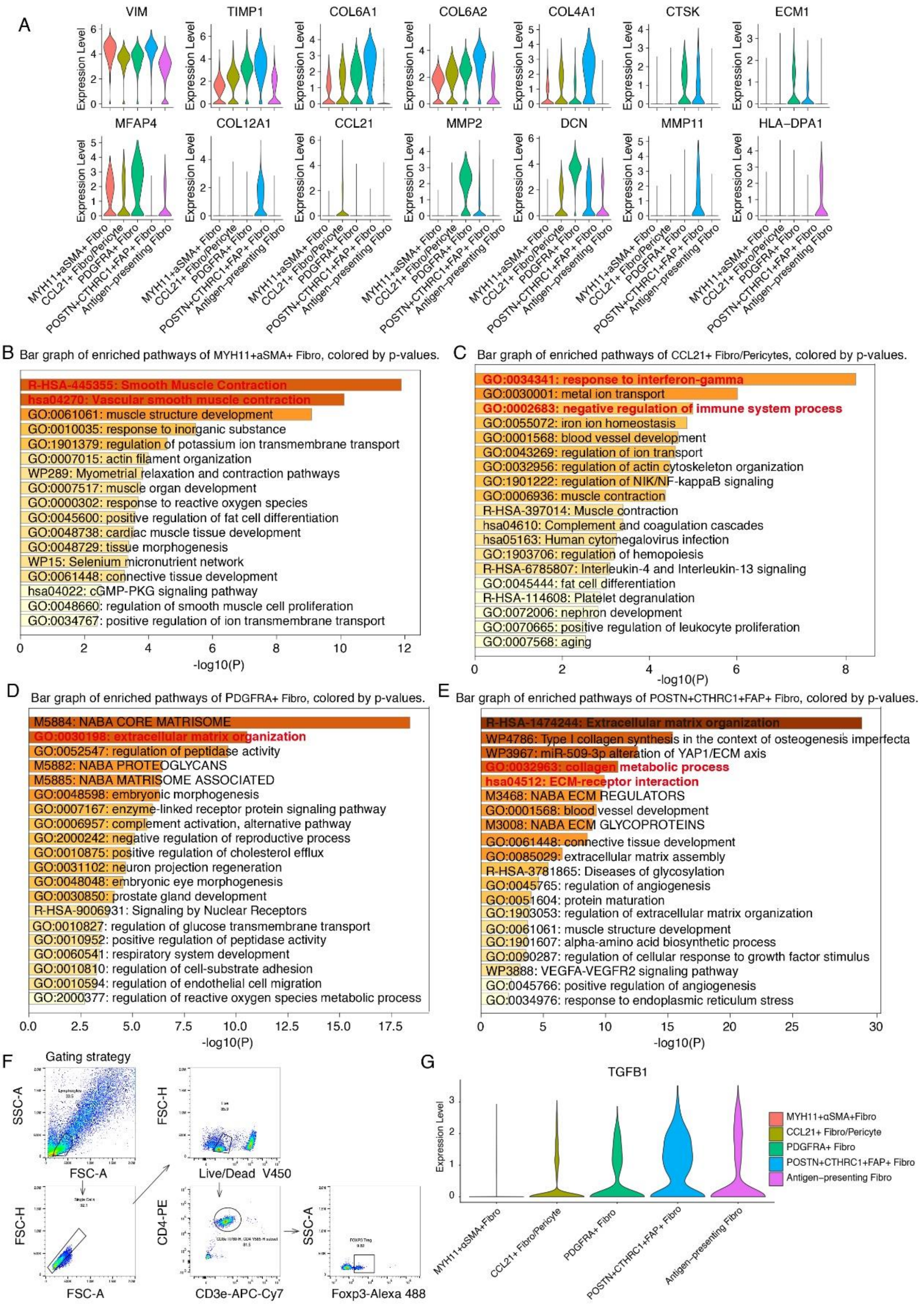

Figure S13. FOXP3^{+}Treg gating strategy of flow cytometry and TGFB expression levels in fibroblast subsets

A. Marker genes expression pattern among the 5 distinct fibroblast subpopulations in violin plot.

B-E. Pathway enrichment analysis suggested the fibroblast subpopulations display contrasting functions in PCa. Extracellular matrix organization (ECM) pathway is top-ranked pathway in POSTN$^+$CTHRC1$^+$FAP$^+$ Fibro subset. In contrast, the MYH11$^+$a-SMA$^+$ Fibro mainly associated with smooth muscle contraction, while genes in CCL21$^+$Fibro/Pericytes mainly associated with response to interferon-gamma and negative regulation of immune system process, respectively. All gene ontology (GO) analyses were performed with the online tool–Metascape (http://metascape.org)[1].

F. Flow cytometry gating strategy for CD4$^+$FoxP3$^+$ regulatory T cells. After gating out doublets (FSC-A/FSC-H) and gating on live (Live/Dead) cells, then the next gate on CD3e and CD4.

G. The POSTN$^+$CTHRC1$^+$FAP$^+$ Fibro subset with the most abundant TGFB expression compared with other fibroblast subsets.

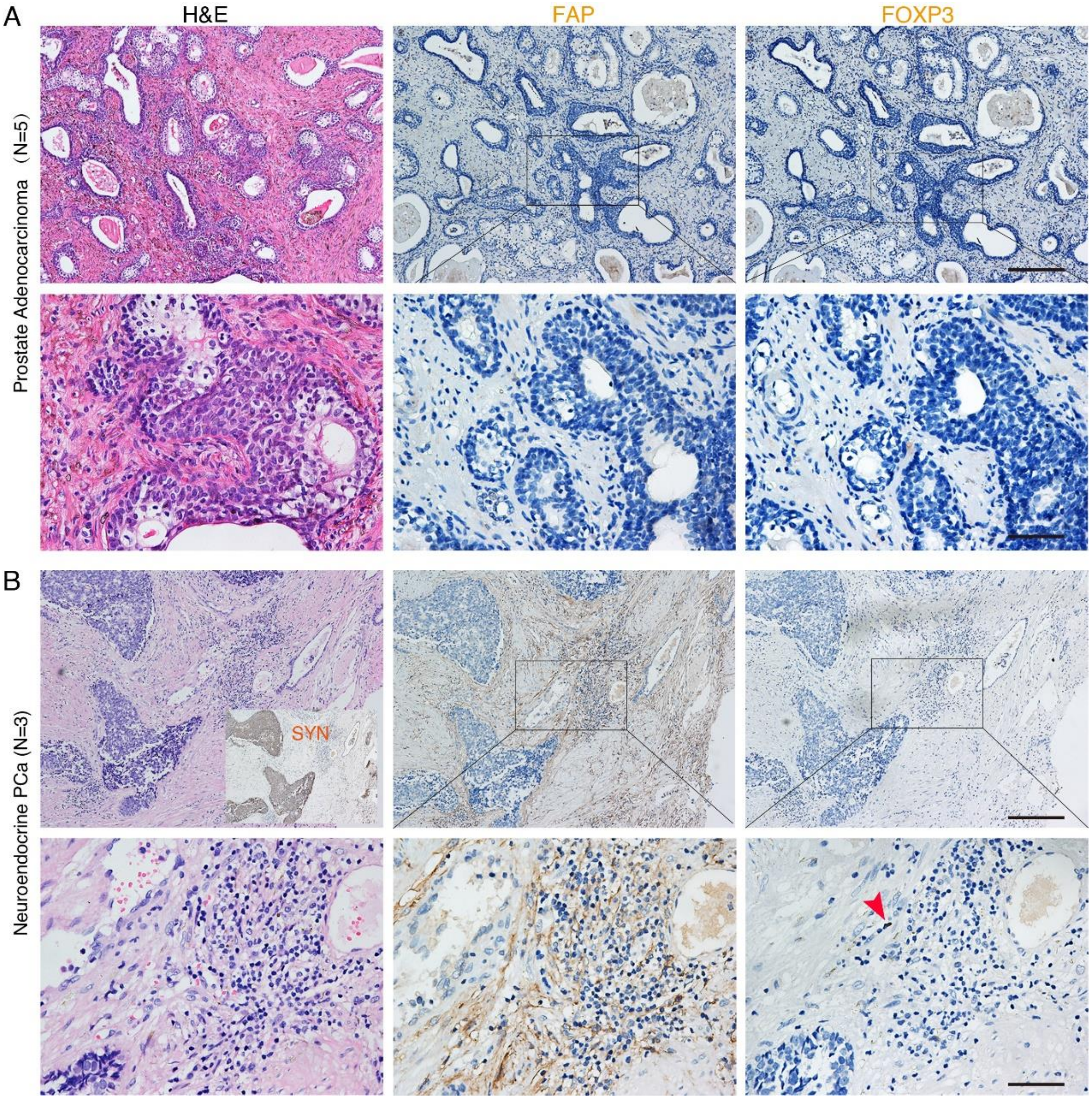

Figure S14. Performing Hematoxylin and Eosin (H&E) staining in combination with immunohistochemistry (IHC) staining to visualize and study the spatial distribution FAP^+ fibroblasts and $FOXP3^+$ regulatory T cells within prostate acinar adenocarcinoma (A) or neuroendocrine prostate cancer samples (B). Anti-FAP antibody for labeling fibroblasts, Anti-FOXP3 antibody for labeling regulatory T cells. Synaptophysin /SYN was utilized as the indicator for neuroendocrine prostate cancer. Scale bar: 200μm. (upper panel), 50μm (lower panel).

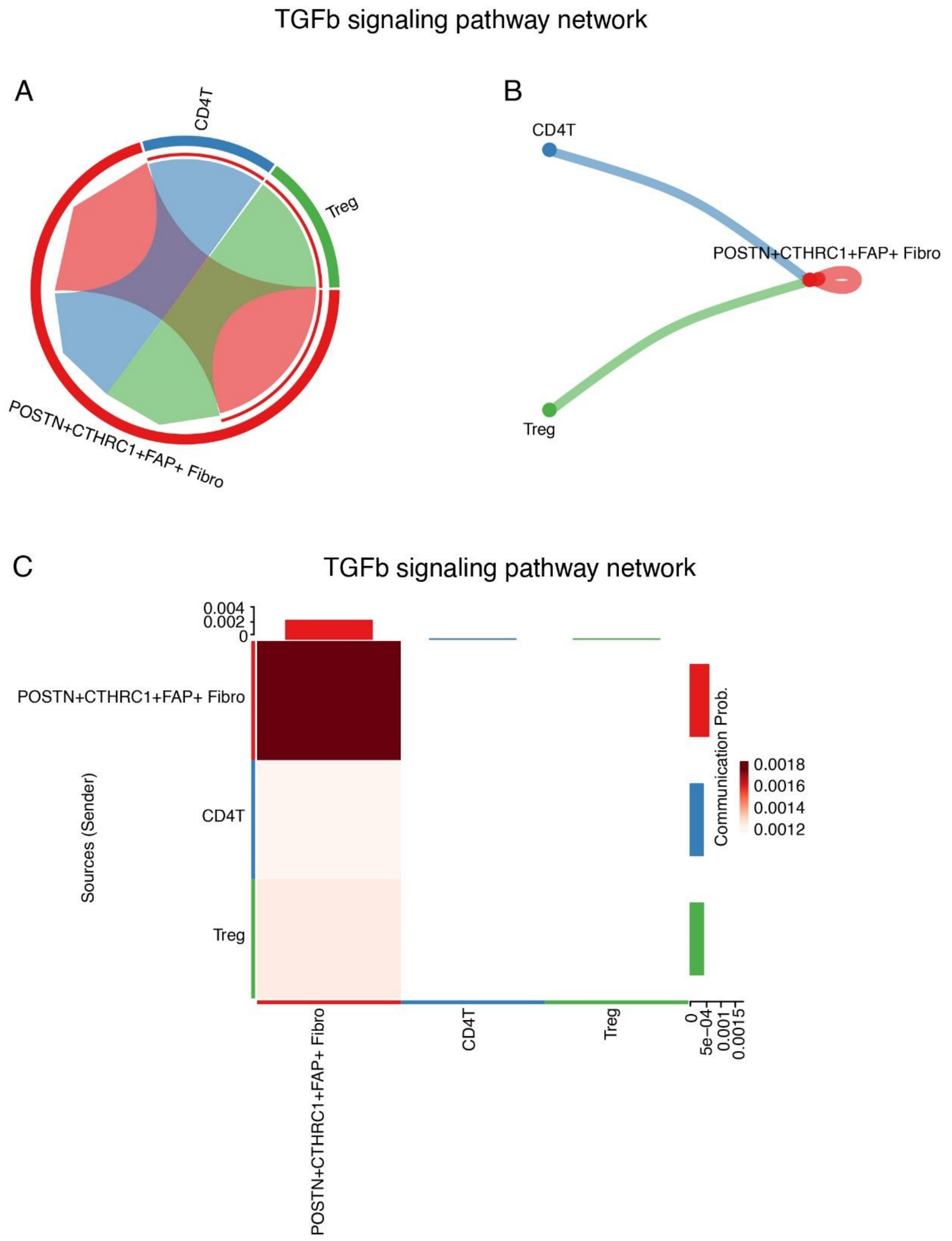

Figure S15. Cell-cell communication among the POSTN+CTHRC1+FAP+ Fibroblasts, CD4T and Treg cells through TGF-β signaling.

A. Illustrate the TGF-β signaling communication using the CellChat:: NetVisual_aggregate layout = "chord"; NetVisual_aggregate layout = "circle"(B); as well as the netVisual_heatmap (C).

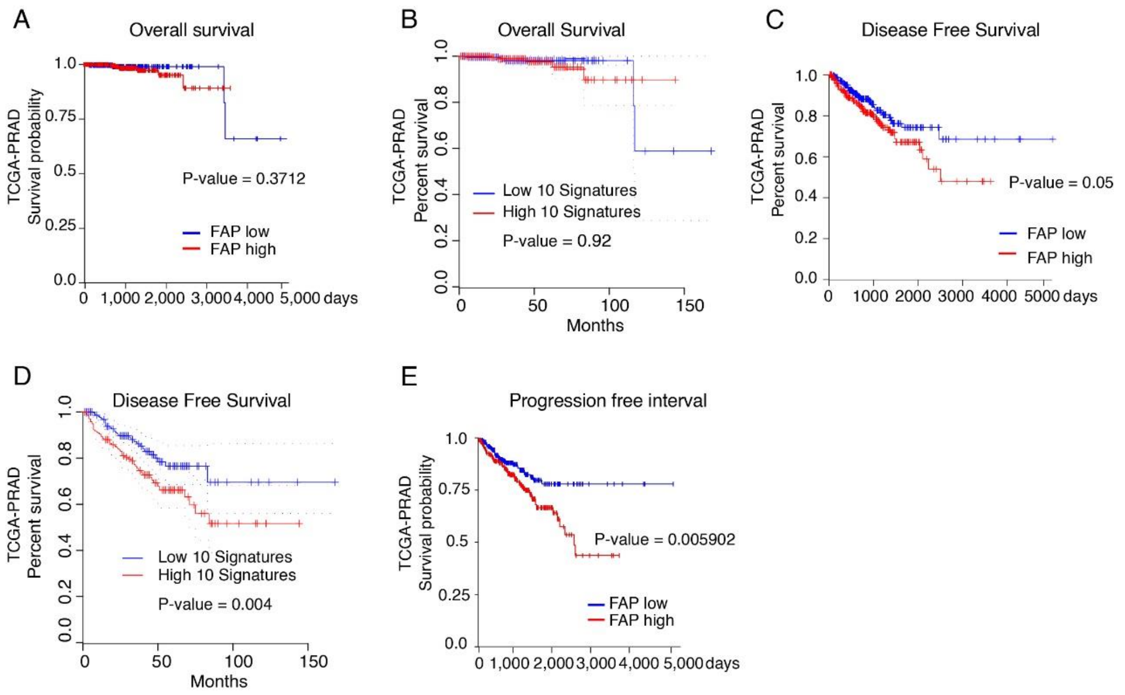

Figure S16. FAP+ fibroblasts may contribute to the PCa progression.

A-D. Utilize the top 10 genes expressed (*POSTN*, *CTHRC1*, *GRP*, *LOXL2*, *LOX*, *COL11A1*, *FAP*, *DUXAP8*, *TNFAIP6*, *COL5A1*) in FAP^{+} fibroblasts as the gene signatures as well as the *FAP* itself to reassess the relationship between the gene expression level with the disease prognosis. Although the OS does not show a significant difference (A & B), DFS using the top 10 gene signatures was more significantly compared to only using the single *FAP* gene (P = 0.004 vs P = 0.05, C & D). Compared to FAP^{high} cases, FAP^{low} patients displayed a longer progression-free interval (PFI, E) in the TCGA-PRAD cohort.

Reference

1 Zhou, Y. *et al.* Metascape provides a biologist-oriented resource for the analysis of systems-level datasets. *Nat Commun* **10**, 1523, doi:10.1038/s41467-019-09234-6 (2019).

Table S1. patient's information

Sample	Patient ID	Gleason Score	Age	Pathological diagnosis	Prior therapy	TNM
1	PCa-1	N.A.	68	neuroendocrine prostate cancer	No	Incomplete information
2	PCa-2	5+4	67	prostate adenocarcinoma	No	T4N1M0
3	PCa-3	4+4	75		No	T4NXM0
4	PCa-4	4+5	64		Abiraterone+ADT	T4NXM1
5	PCa-5	3+3	54		No	T2N0M0
6	PCa-6	3+4	77		No	T4NXM0
7	PCa-7	4+5	74	Castration-resistant prostate cancer	Bicalutamide+ADT	T3N1M0
8	PCa-8	N.A.	70	Normal prostate (dissected from bladder cancer patient)	N.A.	N.A.

N.A.: not available

Table S2. patient's information for club cell				
Group	No.	Age (year)	ADT exposure time	Gleason Score
Hormonally Intact	#1	60	N.A.	3+4
	#2	73		3+4
	#3	64		3+3
	#4	76		4+4
	#5	60		3+4
	#6	57		4+3
	#7	62		4+3
	#8	72		4+5
	#9	69		4+3
	#10	65		4+4
	#11	58		4+4
ADT-treated	#1	66	~ 7 months	4+4
	#2	65	~ 14 months	4+4
	#3	65	~ 8 months	4+3
	#4	55	~ 4 months	4+5
	#5	67	~ 8 weeks	3+4
	#6	70	~ 8 weeks	4+4
	#7	61	~ 8 weeks	4+5
	#8	73	~ 8 weeks	3+3
	#9	71	~ 8 weeks	4+3
	#10	66	~ 4 months	4+5
	#11	76	~ 7 months	4+5
	#12	68	~ 6 months	4+5
	#13	63	~ 3 months	4+5
	#14	66	~ 4 months	5+4
	#15	55	~ 1 week	3+4
	#16	75	~ 2 months	3+3

N.A.: not available
